# Boosting the sonodynamic performance of CoBiMn-layered double hydroxide nanoparticles via tumor microenvironment regulation for ultrasound imaging-guided sonodynamic therapy

**DOI:** 10.1186/s12951-024-02591-5

**Published:** 2024-06-08

**Authors:** Shuqing Yang, Tingting Hu, Gareth R. Williams, Yu Yang, Susu Zhang, Jiayi Shen, Minjiang Chen, Ruizheng Liang, Lingchun Lyu

**Affiliations:** 1 State Key Laboratory of Chemical Resource Engineering, Beijing Advanced Innovation Center for Soft Matter Science and Engineering, Beijing University of Chemical Technology, Beijing, 100029 P. R. China; 2 Department Electrical and Electronic Engineering, The University of Hong Kong, Pokfulam Road, Hong Kong SAR, 999077 P. R. China; 3 UCL School of Pharmacy, University College London, 29-39 Brunswick Square, London, WC1N 1AX UK; 4 Lishui Central Hospital and the Fifth Affiliated Hospital of Wenzhou Medical University, Lishui, 323000 P. R. China; 5 Quzhou Institute for Innovation in Resource Chemical Engineering, Quzhou, 324000 P. R. China

**Keywords:** Layered double hydroxides, Sonosensitizers, Tumor microenvironment, US imaging, Sonodynamic therapy

## Abstract

Sonodynamic therapy (SDT), a promising strategy for cancer treatment with the ability for deep tissue penetration, has received widespread attention in recent years. Sonosensitizers with intrinsic characteristics for tumor-specific curative effects, tumor microenvironment (TME) regulation and tumor diagnosis are in high demand. Herein, amorphous CoBiMn-layered double hydroxide (a-CoBiMn-LDH) nanoparticles are presented as multifunctional sonosensitizers to trigger reactive oxygen species (ROS) generation for ultrasound (US) imaging-guided SDT. Hydrothermal-synthesized CoBiMn-LDH nanoparticles are etched *via* a simple acid treatment to obtain a-CoBiMn-LDH nanoparticles with abundant defects. The a-CoBiMn-LDH nanoparticles give greater ROS generation upon US irradiation, reaching levels ~ 3.3 times and ~ 8.2 times those of the crystalline CoBiMn-LDH nanoparticles and commercial TiO_2_ sonosensitizer, respectively. This excellent US-triggered ROS generation performance can be attributed to the defect-induced narrow band gap and promoted electrons and holes (e^−^/h^+^) separation. More importantly, the presence of Mn^4+^ enables the a-CoBiMn-LDH nanoparticles to regulate the TME by decomposing H_2_O_2_ into O_2_ for hypoxia relief and US imaging, and consuming glutathione (GSH) for protection against ROS clearance. Biological mechanism analysis shows that a-CoBiMn-LDH nanoparticles modified with polyethylene glycol can serve as a multifunctional sonosensitizer to effectively kill cancer cells in vitro and eliminate tumors in vivo under US irradiation by activating p53, apoptosis, and oxidative phosphorylation-related signaling pathways.

**Supplementary Information:**

The online version contains supplementary material available at 10.1186/s12951-024-02591-5.

## Introduction

Ultrasound (US), as a mechanical wave with a high penetration depth over 10 cm in soft tissue, can trigger sonosensitizers to activate the generation of reactive oxygen species (ROS), thereby killing malignant tumors, known as sonodynamic therapy (SDT) [1, 2]. SDT has received tremendous attention because of its deep tissue-penetrating ability, controllability and low skin sensitivity [3, 4]. Effective SDT requires a high concentration of ROS to be generated by sonosensitizers under US irradiation, to induce cell damage [5–7]. Recently, diverse inorganic sonosensitizers with controllable physiochemical properties, high chemical stability and favorable pharmacokinetics for SDT have emerged, such as MnWO_*x*_ nanoparticles [8], Ti_3_C_2_/CuO_2_ nanosheets [9], MoS_2_ nanosheets [10], TiO_1 + x_ nanorods [11], stanene nanosheets [12], and Janus Au-MnO nanoparticles [13]. However, most of the reported inorganic sonosensitizers are restricted by unsatisfactory ROS yields, due to the rapid recombination of US-triggered electrons and holes (e^−^/h^+^) [14–16]. Moreover, the hypoxic level (< 0.1 mM O_2_) and high expression of glutathione (GSH, 1 ~ 10 mM) in the tumor microenvironment (TME) are natural barriers for ROS-mediated anti-tumor therapies. Specifically, the insufficient O_2_ supply in the TME is not conducive to the efficient generation of singlet oxygen (^1^O_2_) by O_2_-dependent sonosensitizers during the SDT process [17], which can also exacerbate tumor growth and metastasis. Meanwhile, the overexpression of GSH in the TME can clear ROS and impair the SDT effect, greatly hindering its implementation in the clinic [18, 19]. To address the above dilemma, several strategies have been proposed for regulation of the TME. For example, Wu et al. fabricated a biodegradable sonosensitizer by encapsulating catalase into silica nanoparticles and then loading with indocyanine green for O_2_ self-supplying SDT [20]. However, these strategies usually involve the integration of multiple components and the preparation process is relatively complex. In addition, with the advancement of US technology, the development of sonosensitizers with contrast capability for imaging-guided SDT is also much sought after [21–23]. Therefore, there is a pressing need to construct single component inorganic sonosensitizers with high ROS generation efficiency, O_2_-generating ability, GSH consumption, and imaging contrast capability.

Layered double hydroxides (LDHs) are characterized by tunable chemical composition/ structure, pH-responsive degradation, and high biocompatibility [24–26]. They have been extensively explored in a variety of biomedical applications, including cancer therapy, diagnostic imaging, anti-bacterial formulations, biosensing, and tissue engineering [27–29]. For example, Gd-doped MgAl-LDH nanosheets co-loaded with doxorubicin and indocyanine green can achieve synergistic chemotherapy and photothermal therapy [30]. Isophthalic acid-intercalated ZnAl-LDHs with high ^1^O_2_ quantum yields can function as photosensitizers for near infrared (NIR) photodynamic therapy [31]. There have also been reports on the use of LDHs as sonosensitizers for SDT. For instance, CoW-LDH nanosheets prepared through a crystalline-to-amorphous phase transformation strategy have been proven to be a high-efficiency sonosensitizer for SDT [32]. However, currently reported LDH-based sonosensitizers are relatively monofunctional and cannot adequately regulate the TME to maximize ROS production. Therefore, developing a new LDH-based sonosensitizer with high ROS generation activity, TME remodeling ability and multifunctionality is of great significance for expanding the application of LDH-based nanomaterials in the field of SDT.

**Fig. 1:**
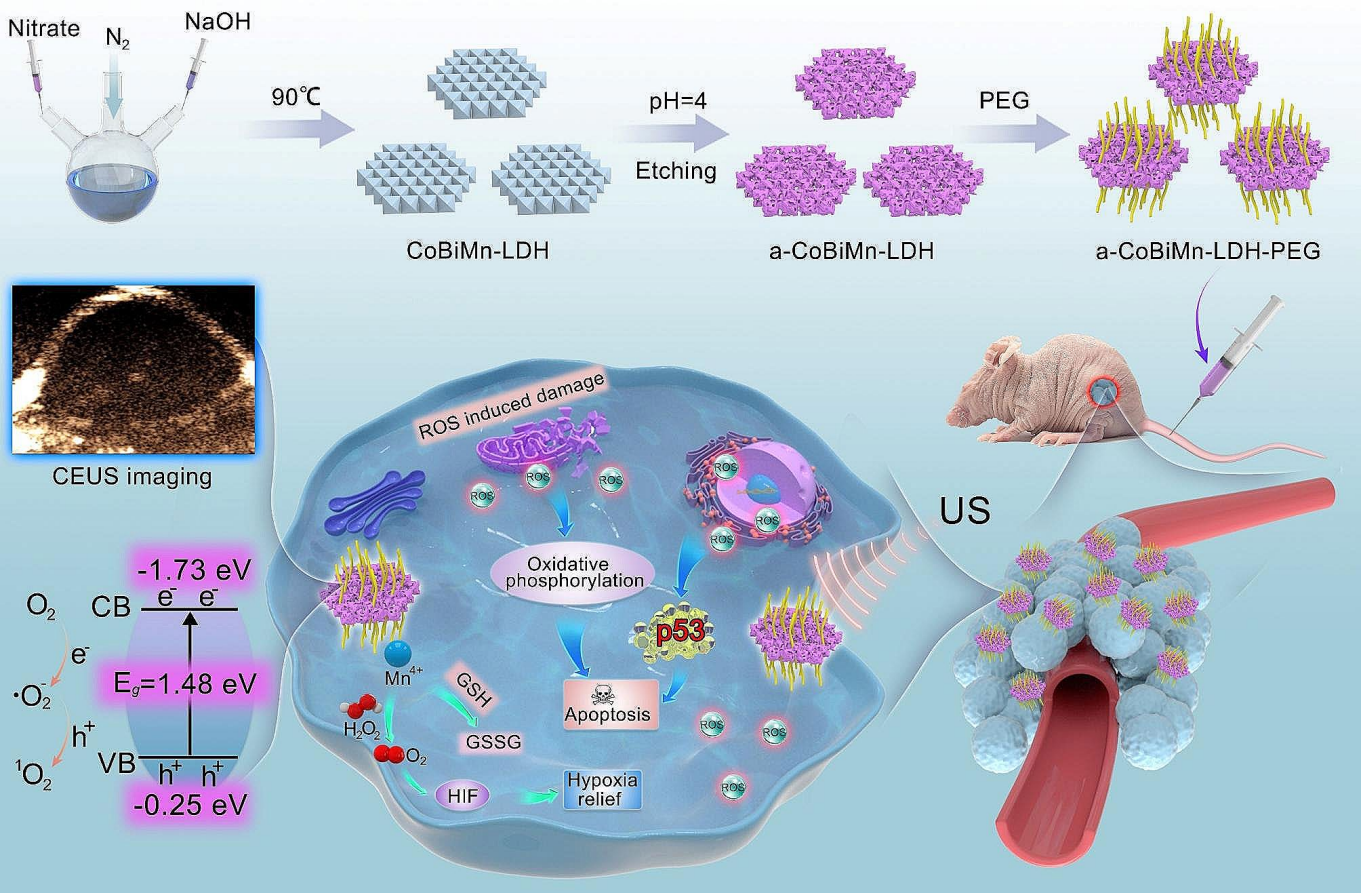
Schematic illustration of the preparation of a-CoBiMn-LDH-PEG nanoparticles and their application in US-imaging guided SDT

It is well known that Mn^4+^ can catalyze the reaction of H_2_O_2_ to produce O_2_ in an acidic environment, and also has oxidation activity to consume GSH [33–35]. Herein, we report the design of amorphous CoBiMn-LDH (a-CoBiMn-LDH) nanoparticles *via* an acid etching strategy, and explore these as a multifunctional sonosensitizer for US imaging-guided SDT (Fig. 1). Hydrothermal-synthesized CoBiMn-LDH nanoparticles with high crystallinity can be transformed into an amorphous phase after acid etching, which is accompanied by the generation of abundant defects. The a-CoBiMn-LDH nanoparticles display superior activity in terms of ROS generation upon US irradiation, which is ~ 3.3 times and ~ 8.2 times that of the crystalline CoBiMn-LDH nanoparticles and commercial TiO_2_ sonosensitizer, respectively. Our results indicate that the high ROS efficiency of a-CoBiMn-LDH could be attributed to the defect-induced narrow band gap and promoted e^−^/h^+^ separation. More importantly, the existence of Mn^4+^ endows the a-CoBiMn-LDH nanoparticles with the ability to regulate the TME by decomposing H_2_O_2_ into O_2_ for hypoxia relief and US imaging, and consuming GSH for protection against ROS clearance. In vitro and in vivo experiments suggest that a-CoBiMn-LDH nanoparticles modified with polyethylene glycol (PEG) exhibit excellent SDT performance, effectively killing cancer cells and eliminating tumors under US irradiation by activating signaling pathways such as p53, apoptosis, and oxidative phosphorylation. Our work indicates that a-CoBiMn-LDH nanoparticles can function as a highly active sonosensitizer with multifunctionality for US imaging-guided SDT, which is conducive to tumor diagnosis and therapeutic effect monitoring, thereby improving the accuracy and efficiency of cancer treatment.

## Results and discussion

### Material synthesis and characterization

**Fig. 2:**
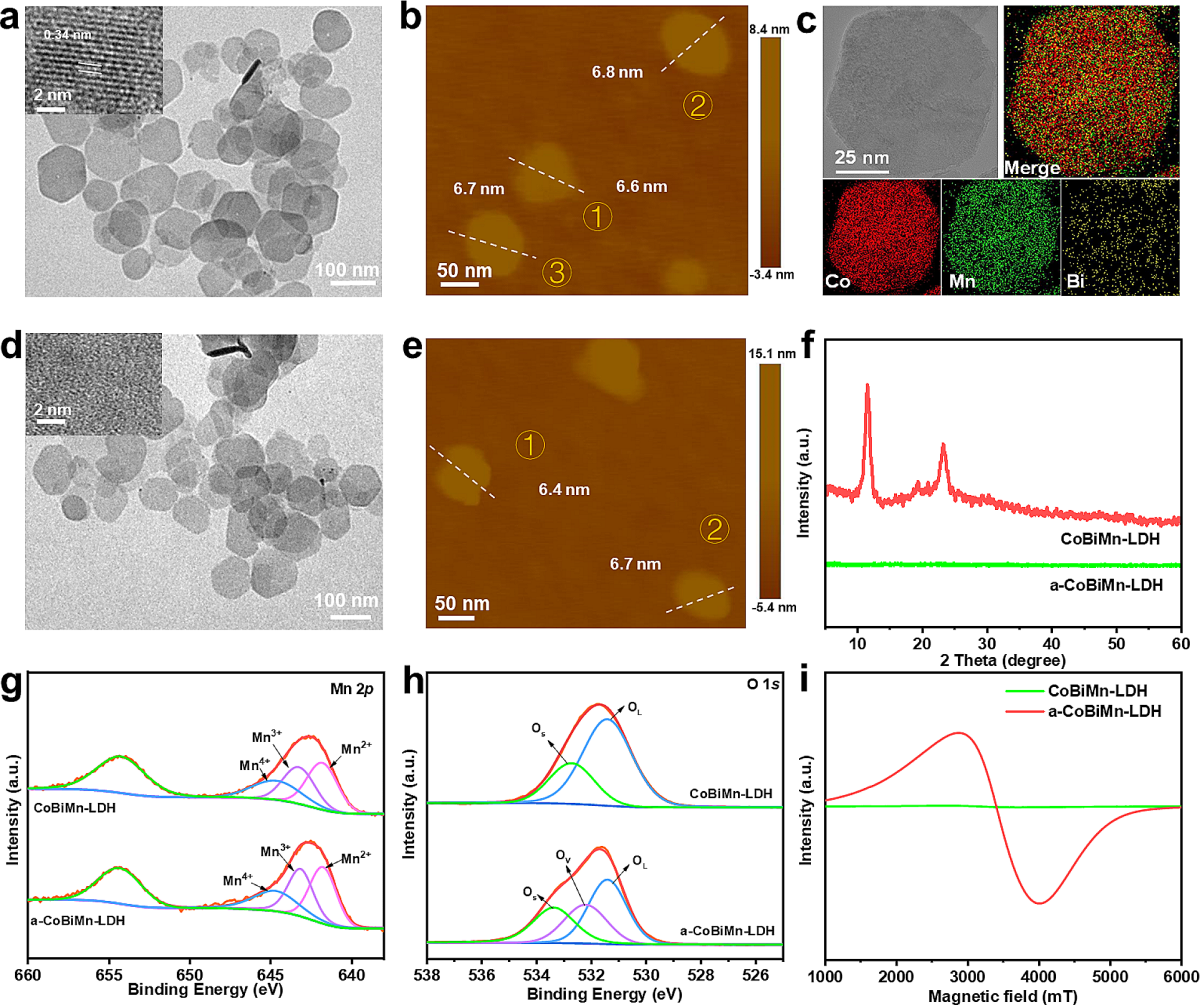
(**a**) HR-TEM, (**b**) AFM and (**c**) EDX mapping images of CoBiMn-LDH nanoparticles. (**d**) HR-TEM and (**e**) AFM images of a-CoBiMn-LDH nanoparticles. (**f**) XRD patterns of CoBiMn-LDH and a-CoBiMn-LDH nanoparticles. (**g**) Mn 2p and (**g**) O 1s XPS spectra of CoBiMn-LDH and a-CoBiMn-LDH nanoparticles. (i) ESR spectra of CoBiMn-LDH and a-CoBiMn-LDH nanoparticles

Firstly, we synthesized CoBiMn-LDH nanoparticles doped with different Bi content by a wet-chemical method. X-ray diffraction (XRD) patterns showed the characteristic (003) and (006) diffraction peaks of CoBiMn-LDH (Fig. S1) [36, 37], proving the successful synthesis of LDH materials. High-resolution transmission electron microscopy (HR-TEM) images revealed that the size of the CoBiMn-LDH nanoparticles is 60–110 nm with the lattice spacing of ~ 0.34 nm (Fig. 2a), which corresponds to the (006) plane of the LDH crystal structure [38]. From the atomic force microscopy (AFM) image, the thickness of CoBiMn-LDH nanoparticles is found to be 6–7 nm (Fig. 2b and S2a). Energy-dispersive X-ray (EDX) elemental mapping indicated a uniform distribution of Co, Bi and Mn in the nanoparticles (Fig. 2c). After acid etching, the size of the obtained a-CoBiMn-LDH nanoparticles is slightly decreased and no obvious lattice was observed by HR-TEM (Fig. 2d), indicating its amorphous structure. As revealed by the AFM image, there was no notable change in the thickness of the a-CoBiMn-LDH nanoparticles after acid treatment (Fig. 2e and S2b). In addition, compared with CoBiMn-LDH, no Bragg reflections are observed in the XRD pattern of a-CoBiMn-LDH (Fig. 2f), further demonstrating that the a-CoBiMn-LDH nanoparticles are transformed from crystalline to amorphous [39].

To investigate the structural differences before and after acid etching, X-ray photoelectron spectroscopy (XPS) analysis was carried out (Fig. S3a and S3b). Co 2p XPS spectra showed the characteristic peaks of Co^2+^ 2p_3/2_, Co^2+^ 2p_1/2_, Co^3+^ 2p_3/2_, Co^3+^ 2p_1/2_ at 782.51, 798.42, 781.11, and 796.60 eV in both the CoBiMn-LDH and a-CoBiMn-LDH nanoparticles (Fig. S3c). Interestingly, the Co^3+^/Co^2+^ ratio of a-CoBiMn-LDH (1.35) is higher than that of CoBiMn-LDH (0.91). In the Bi 4f spectra, the peaks at 159.04 and 164.37 eV in CoBiMn-LDH could be assigned to Bi^3+^ 4f_7/2_ and Bi^3+^ 4f_5/2_ (Fig. S3d). These slightly shift to higher binding energy at 159.75 and 165.02 eV in a-CoBiMn-LDH, but in both cases the presence of Bi^3+^ is confirmed. The Mn 2p spectra of CoBiMn-LDH and a-CoBiMn-LDH contain binding energy peaks at 644.68, 643.37 and 641.90 eV (Fig. 2g), which were assigned to Mn^4+^ 2p_3/2_, Mn^3+^ 2p_3/2_ and Mn^2+^ 2p_3/2_, respectively, indicating that Mn^4+^, Mn^3+^ and Mn^2+^ existed simultaneously in the nanoparticles. O 1s spectra (Fig. 2h) reveal that different oxygen species including lattice oxygen (531.46/531.43 eV) and adsorbed oxygen (532.76/533.34 eV) existed in both CoBiMn-LDH and a-CoBiMn-LDH. It is worth pointing out that a peak from oxygen vacancies (OVs, 532.18 eV) existed in a-CoBiMn-LDH, proving the generation of abundant OVs after acid etching [40]. The existence of defects was further investigated by electron spin resonance (ESR) spectroscopy (Fig. 2i). The a-CoBiMn-LDH nanoparticles exhibit an obvious peak at G = 2.1, which is absent in CoBiMn-LDH, confirming the generation of rich defects by the etching process [41].

### SDT performance

**Fig. 3:**
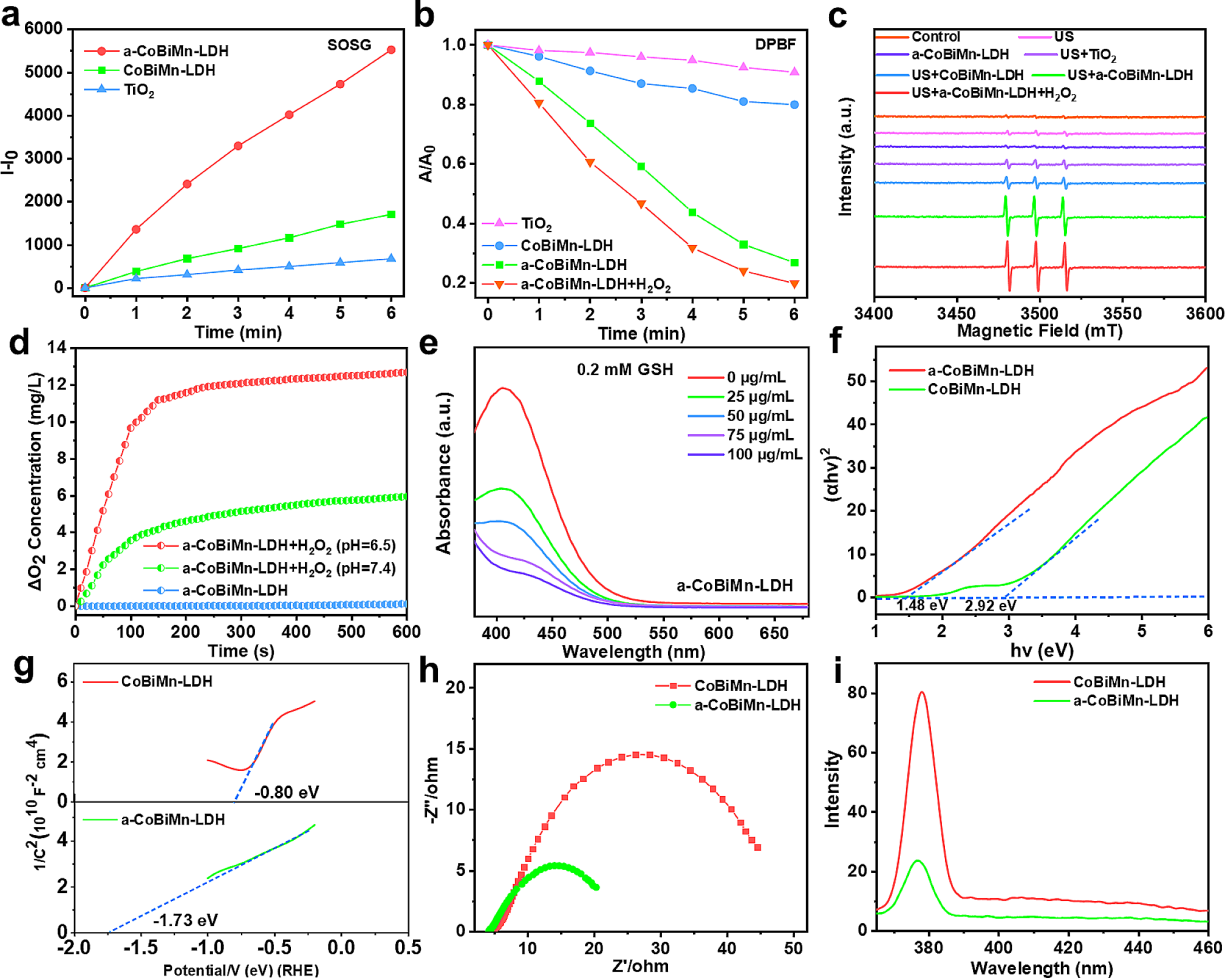
(**a**) Fluorescence intensity of SOSG in the presence of TiO_2_, CoBiMn-LDH and a-CoBiMn-LDH nanoparticles under US irradiation (40 kHz, 3 W cm^− 2^, 6 min). (**b**) Normalized attenuation curves of DPBF in the presence of TiO_2_, CoBiMn-LDH, a-CoBiMn-LDH and a-CoBiMn-LDH + H_2_O_2_ under US irradiation (40 kHz, 3 W cm^− 2^, 6 min). (**c**) ESR spectra of ^1^O_2_ produced under different conditions. (**d**) Oxygen production curves of a-CoBiMn-LDH + H_2_O_2_ at pH = 7.4 and 6.5. (**e**) GSH consumption at different concentrations of a-CoBiMn-LDH (0, 25, 50, 75, 100 µg mL^− 1^). (**f**) Band gaps of CoBiMn-LDH and a-CoBiMn-LDH nanoparticles, and (**f**) their Mott-Schottky diagrams. (**h**) Electrochemical impedance spectra and (**i**) PL spectra of CoBiMn-LDH and a-CoBiMn-LDH nanoparticles

The ability of CoBiMn-LDH and a-CoBiMn-LDH in ROS generation was investigated using singlet oxygen sensor green (SOSG) [42]. After US irradiation for 6 min, the fluorescence intensity of SOSG in the a-CoBiMn-LDH group was significantly stronger (~ 3.3 times) than that of CoBiMn-LDH group (Fig. 3a and S4), indicating that the ROS generation activity could be enhanced by acid etching. The SDT properties of a-CoBiMn-LDH nanoparticles with different Bi content (10%, 20%, 30%) were also explored. It was found that the a-CoBiMn-LDH nanoparticles with 20% Bi content exhibited the strongest ROS generation performance under US irradiation (Fig. S5). In addition, the SDT properties of a-CoBiMn-LDH (if there is no special indication, a-CoBiMn-LDH refers to a-CoBiMn-LDH (20%)) under different pH environments (pH = 5.4, 6.5 and 7.4) were evaluated. As shown in Fig. S6, a-CoBiMn-LDH nanoparticles dispersed in a pH = 6.5 buffer solution (simulated tumor microenvironment) displayed the strongest fluorescence intensity, implying the system should have potent ROS generation performance in the TME. Moreover, we compared the SDT performance of a-CoBiMn-LDH with a commercial TiO_2_ sonosensitizer. In Fig. S4 and 3a, it can be seen that the ROS generation activity of a-CoBiMn-LDH is ~ 8.2 times that of the commercial TiO_2_. 1,3-Diphenylisobenzofuran (DPBF) and ESR assays further verified the superior SDT performance of a-CoBiMn-LDH than CoBiMn-LDH and TiO_2_ (Fig. 3b and c and S7), as the more prominent decrease in absorbance of DPBF and the stronger characteristic signal of ^1^O_2_ (1:1:1) were found in a-CoBiMn-LDH group.

It has been reported that Mn^4+^ can catalyze the reaction of H_2_O_2_ to generate O_2_ [43]. On this basis, the catalytic activity of a-CoBiMn-LDH towards O_2_ generation was investigated by dissolving oxygen equipment. In Fig. 3d, the O_2_ concentration in a suspension of a-CoBiMn-LDH at pH values of 7.4 and 6.5 increased rapidly after the addition of H_2_O_2_, to 5.95 mg L^− 1^ and 12.68 mg L^− 1^, respectively. In contrast, no O_2_ generation was seen in the absence H_2_O_2_, proving the ability of a-CoBiMn-LDH to generate O_2_ by decomposing H_2_O_2_. In order to investigate the effect of O_2_ on ROS generation, DPBF was used as a probe to detect US-triggered ^1^O_2_ generation [44]. It was found that the absorbance of DPBF in the a-CoBiMn-LDH nanoparticles group decreased significantly under US irradiation (Fig. 3b). Interestingly, a more pronounced decline in the absorbance of DPBF was observed after the addition of H_2_O_2_ (Fig. 3b and S4), suggesting that O_2_ generation induced by the reaction between Mn^4+^ and H_2_O_2_ effectively promotes ^1^O_2_ production. The generation of ^1^O_2_ was further investigated by ESR spectroscopy with the 2,2,6,6-tetramethy l-4-piperidone (TEMP) probe. As presented in Fig. 3c, compared with other groups, the a-CoBiMn-LDH + H_2_O_2_ group showed the strongest peaks, further proving that O_2_ generation could promote the SDT performance of a-CoBiMn-LDH. In addition, since Mn^4+^ has been reported to react with reduced GSH, the GSH consumption capacity of a-CoBiMn-LDH nanoparticles was studied using 5,5’-dithiobis-2-nitrobenzoic acid (DTNB). As can be seen from Fig. 3e, the GSH content gradually decreased with an increase of a-CoBiMn-LDH concentration, suggesting that the nanoparticles possess GSH depletion ability. This should be conductive to reducing the clearance of ROS by GSH and promoting SDT performance.

### Mechanism of ROS generation

To reveal the ROS generation mechanism, ultraviolet–visible–near-infrared (UV-vis-NIR) diffuse reflection spectroscopy was conducted to analyze the band structures of CoBiMn-LDH and a-CoBiMn-LDH. As presented in Fig. 3f and S8, the band gaps (*E*_g_) of CoBiMn-LDH and a-CoBiMn-LDH nanoparticles were calculated to be 2.92 eV and 1.48 eV respectively, indicating a decrease in the *E*_g_ after acid treatment. The conduction band (CB) positions were determined using Mott–Schottky plots (Fig. 3g), and the CB potentials of CoBiMn-LDH and a-CoBiMn-LDH nanoparticles were measured to be − 0.80 and − 1.73 eV. Accordingly, their valence band (VB) potentials were determined to be 2.12 and − 0.25 eV. The lower CB and VB values of a-CoBiMn-LDH nanoparticles were beneficial for the excitation of e^−^ and h^+^ and significantly improved their separation, with e^−^ and h^+^ occupying CB and VB respectively [45]. The energy level diagram and e^−^ transfer processes for CoBiMn-LDH and a-CoBiMn-LDH are shown in Fig. S9. Under US irradiation, the excited e^−^ first reacts with O_2_ to form the intermediate ·O_2_^−^, which further combines with h^+^ to produce the final ^1^O_2_ [46]. Dihydrorhodamine 123 (DHR 123) that can be oxidized by ·O_2_^−^ to emit a fluorescence signal at 526 nm was used as a specific probe to detect the intermediate ·O_2_^−^. In Fig. S10, the fluorescence intensity of DHR 123 was weak after the addition of CoBiMn-LDH nanoparticles, while the fluorescence intensity in the a-CoBiMn-LDH group was significantly enhanced, indicating the superior capacity of a-CoBiMn-LDH to generate ·O_2_^−^.

Electrochemical impedance spectroscopy (EIS) was utilized to investigate the e^−^-h^+^ separation ability of CoBiMn-LDH nanoparticles, where a smaller radius of the Nyquist circle means a faster electron transfer rate [47, 48]. As shown in Fig. 3h, the radius of a-CoBiMn-LDH nanoparticles in the EIS Nyquist diagram is much smaller than that of CoBiMn-LDH nanoparticles, demonstrating faster charge transfer occurred on the interface of the a-CoBiMn-LDH nanoparticles electrode. Photoluminescence spectroscopy (PL) is also an effective way to demonstrate the efficiency of e^−^-h^+^ recombination [49]. In Fig. 3i, the CoBiMn-LDH nanoparticles exhibit a strong emission peak at 371 nm. However, the peak intensity of a-CoBiMn-LDH was much weaker than that of CoBiMn-LDH, meaning that the radiative recombination of e^−^ and h^+^ in a-CoBiMn-LDH was significantly inhibited, beneficial for the promotion of ROS generation.

### Surface modification

The aforementioned results demonstrate the potent activity of a-CoBiMn-LDH as a sonosensitizer. Next, PEG modification was performed on a-CoBiMn-LDH nanoparticles to improve its biocompatibility [50]. In Fourier transform infrared spectroscopy (FT-IR), the characteristic peak of LDH at 1380 cm^− 1^ (N-O vibration of nitrate) and the characteristic bands of PEG at 950 cm^− 1^ (C–O–C vibration) and 846 cm^− 1^ (− CH_2_ − in-plane rocking) were found in a-CoBiMn-LDH-PEG (Fig. S11), indicating successful PEGylation. Zeta potential analysis was also conducted to verify this result. As presented in Fig. S12, the zeta potential of a-CoBiMn-LDH decreased from 13.8 ± 1.2 mV to − 10.7 ± 1.1 mV after acid etching. After PEGylation, the zeta potential of a-CoBiMn-LDH-PEG in water was − 13.5 ± 1.0 mV, which is similar to that in PBS (− 14.6 ± 1.3 mV) and high-glucose Dulbecco’s modified Eagles medium (DMEM) (− 14.0 ± 1.1 mV). The interaction between a-CoBiMn-LDH and PEG could be attributed to the Van der Waals’ force and hydrogen bonding [39]. Dynamic light scattering (DLS) analysis showed that the hydrodynamic size of a-CoBiMn-LDH-PEG is 105.1 ± 2.2 nm, larger than that of CoBiMn-LDH (90.5 ± 2.6 nm) and a-CoBiMn-LDH (78.8 ± 1.8 nm) (Fig. S13a). There was no observable size variation of a-CoBiMn-LDH-PEG after suspension in water, PBS or DMEM for one week (Fig. S13b), indicating good stability.

### Evaluation of *in vitro* therapeutic effect on 4T1 cells.

**Fig. 4:**
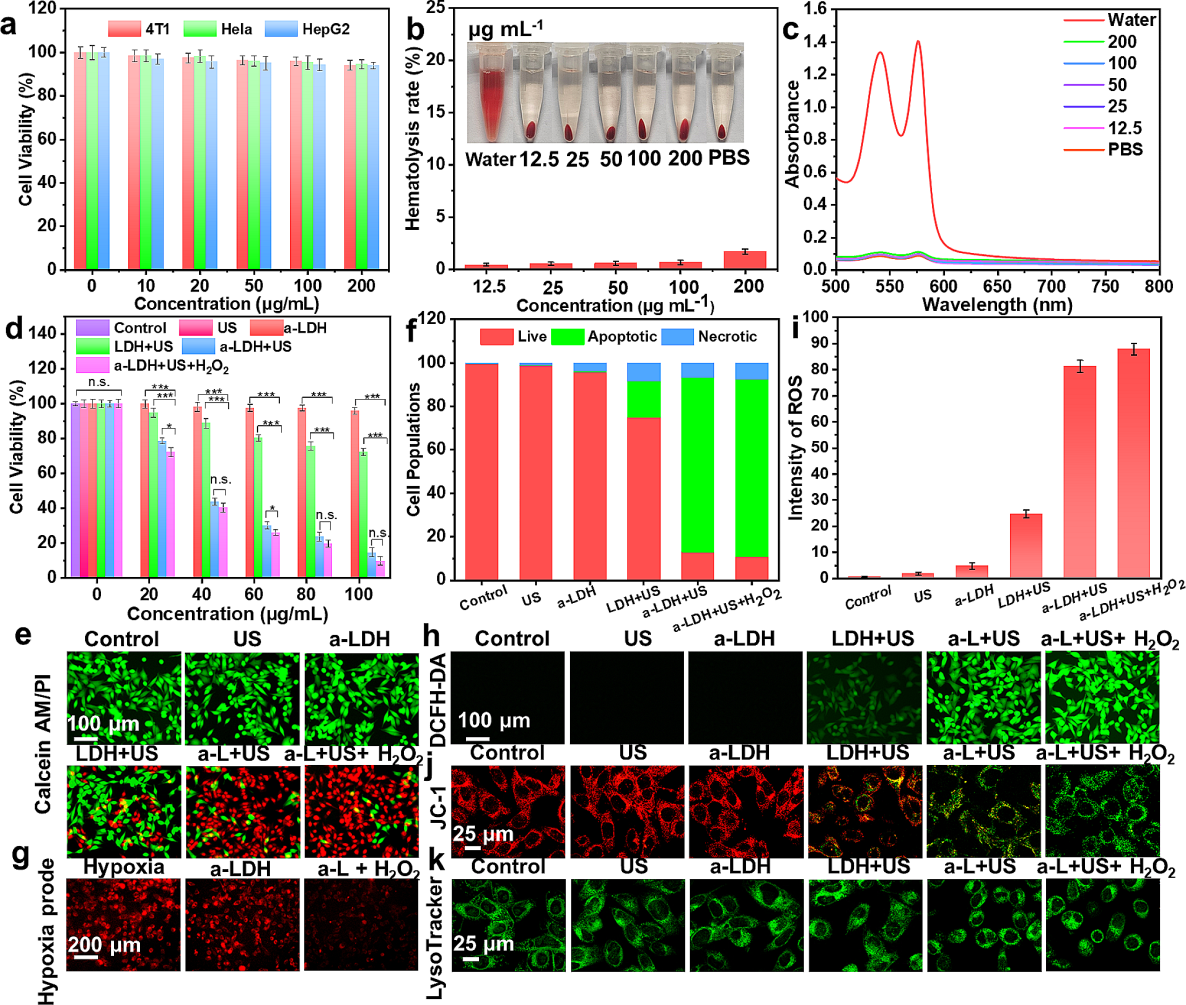
(**a**) Cell viability of 4T1, Hela and HepG2 cells cultured with a-CoBiMn-LDH-PEG at different concentrations. (**b**) Hemolysis rate of red blood cells after incubation with different concentrations of a-CoBiMn-LDH-PEG nanoparticles (12.5, 25, 50, 100, 200 µg mL^− 1^). Inset: representative photographs. (**c**) Absorbance of red blood cell supernatant treated with water, PBS and a-CoBiMn-LDH-PEG nanoparticles at different concentrations. (**d**) Cell viability of 4T1 cells under different conditions: (1) control, (2) US (40 kHz, 3 W cm^− 2^, 6 min), (3) a-CoBiMn-LDH-PEG, (4) CoBiMn-LDH-PEG + US, (5) a-CoBiMn-LDH-PEG + US, (6) a-CoBiMn-LDH-PEG + US + H_2_O_2_, and (**e**) corresponding Calcein-AM/PI staining images. (**f**) Quantitative apoptosis analysis of 4T1 cells after Annexin V-FITC/PI co-staining. (**g**) [Ru(dpp)_3_]Cl_2_ staining images under hypoxic conditions. (**h**) DCFH-DA staining images and (**i**) corresponding ROS quantitative analysis. (**j**) JC-1 and (**k**) LysoTracker Green staining images of 4T1 cells after different treatments. Data are expressed as mean ± S.D (*n* = 3). *p < 0.05, **p < 0.01, ***p < 0.001

The SDT-mediated therapeutic performance of a-CoBiMn-LDH-PEG was next evaluated in vitro. Firstly, the cellular uptake of a-CoBiMn-LDH-PEG nanoparticles by 4T1 cells was studied [51]. In Fig. S14, a strong green fluorescence signal from FITC-labeled a-CoBiMn-LDH-PEG nanoparticles was seen in cells over 24 h of incubation. Thus, a-CoBiMn-LDH-PEG nanoparticles could be effectively internalized by cells. The biocompatibility of a-CoBiMn-LDH-PEG nanoparticles on 4T1, Hela and HepG2 cells was measured using standard methyl thiazolyl tetrazolium (MTT) assays. The cytotoxicity of a-CoBiMn-LDH-PEG nanoparticles to cells was found to be negligible even at a high concentration of 200 µg mL^− 1^ (Fig. 4a), suggesting satisfactory biocompatibility. The biosafety of a-CoBiMn-LDH-PEG nanoparticles was further evaluated with a hemolysis assay [52]. In Fig. 4b and c, it can be seen that after incubation with various concentrations (12.5 to 200 µg mL^− 1^) of a-CoBiMn-LDH-PEG nanoparticles, the hemolysis rate was always lower than 5% (standard values), and thus the system does not cause significant hemolysis.

Given the apparently biosafety of the formulations, the therapeutic effect of a-CoBiMn-LDH-PEG was investigated with 4T1 cells (Fig. 4d). The control, US alone and a-CoBiMn-LDH-PEG alone groups led to negligible cytotoxicity, while the cell viability of CoBiMn-LDH-PEG + US and a-CoBiMn-LDH-PEG + US groups significantly decreased to 72.3% and 14.8% at a concentration of 100 µg mL^− 1^, respectively. Moreover, the addition of H_2_O_2_ further enhanced the cytotoxic effect of a-CoBiMn-LDH-PEG under US irradiation, resulting in a cell viability of 9.8% and demonstrating the strong killing effect on 4T1 cells of a-CoBiMn-LDH-PEG in the presence of H_2_O_2_. Live (calcein acetoxymethyl ester, Calcein-AM)/dead (propidium iodide, PI) double staining analysis also evidenced the efficiency of a-CoBiMn-LDH-PEG nanoparticles for in vitro SDT. The results in Fig. 4e and S15 reveal no red fluorescence of PI (denoting dead cells) in the control, US and a-CoBiMn-LDH-PEG groups, while obvious red fluorescence was present in the CoBiMn-LDH-PEG + US and a-CoBiMn-LDH-PEG + US groups. Moreover, the brightest red fluorescence was observed in the a-CoBiMn-LDH-PEG + US + H_2_O_2_ group, consistent with the highest ROS generation efficiency being with the a-CoBiMn-LDH-PEG nanoparticles in the presence of H_2_O_2_ under US irradiation. Flow cytometry further confirmed that a-CoBiMn-LDH-PEG + US and a-CoBiMn-LDH-PEG + US + H_2_O_2_ groups successfully induced SDT-related apoptosis (Fig. 4f and S16).

Since a-CoBiMn-LDH possessed catalytic activity towards O_2_ generation, the hypoxia level of 4T1 cells was monitored using a red hypoxia staining reagent ([Ru(dpp)_3_]Cl_2_) [53]. Strong fluorescence signals were observed in the blank and a-CoBiMn-LDH-PEG groups, while the fluorescence intensity in the a-CoBiMn-LDH-PEG + H_2_O_2_ group decreased significantly (Fig. 4g), demonstrating that a-CoBiMn-LDH-PEG nanoparticles could alleviate hypoxia in the presence of H_2_O_2_, due to O_2_ generation. Furthermore, intracellular ROS levels were detected with the 2’,7’-dichlorofluorescein diacetate (DCFH-DA) probe. In Fig. 4h, no 2’,7’-dichlorofluorescein (DCF) fluorescence was observed in cells treated with DMEM, a-CoBiMn-LDH-PEG nanoparticles or US irradiation alone. However, under US irradiation, green fluorescence enhancement was induced by CoBiMn-LDH-PEG and a-CoBiMn-LDH-PEG, and the fluorescence intensity was enhanced when H_2_O_2_ was also present. ROS quantitative analysis further verified the above results (Fig. 4i).

Subsequently, the mitochondrial dysfunction of different groups was assessed using 5,5’,6,6’-tetrachloro-1,1’-3,3’-tetraethyl-benzimidazolylcarbocyanine iodide (JC-1), a mitochondrial membrane potential dye (see Fig. 4j and S17). Negligible green fluorescence from JC-1 monomers was observed in the control, US alone and a-CoBiMn-LDH-PEG groups, while increased green fluorescence was visible in the CoBiMn-LDH-PEG + US and a-CoBiMn-LDH-PEG + US groups. This the green fluorescence was significantly enhanced after the addition of H_2_O_2_, indicating extensive mitochondrial damage. We also examined the effects of different treatments on lysosomes, using the LysoTracker Green probe (Fig. 4k) [54]. The cells in the control, US alone, and a-CoBiMn-LDH-PEG groups showed green stain spots due to the lysosome wrapped in the cytoplasm, while the cells treated with CoBiMn-LDH-PEG + US showed blurred green stain spots. In the a-CoBiMn-LDH-PEG + US and a-CoBiMn-LDH-PEG + US + H_2_O_2_ groups, the green spots almost disappeared, indicating serious lysosome damage.

### Biological mechanism analysis

**Fig. 5:**
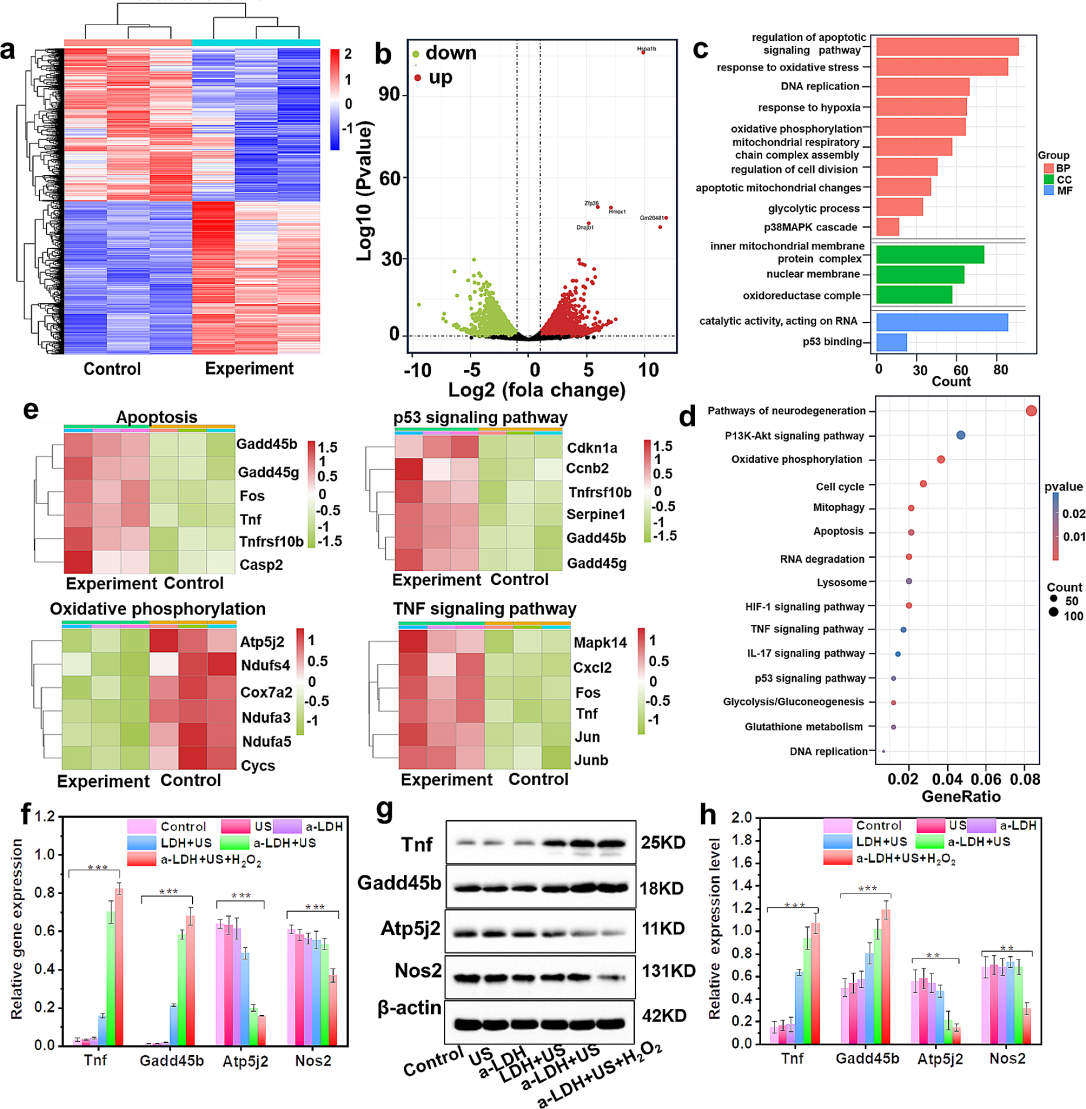
The biological mechanism mediated by a-CoBiMn-LDH-PEG. (**a**) Heat map of differentially expressed genes between the a-CoBiMn-LDH-PEG + H_2_O_2_ + US (experiment) group and control group. (**b**) Volcano plot analysis, (**c**) GO and (**d**) KEGG enrichment analysis. (**e**) Heat map of expressed genes related to p53, oxidative phosphorylation, TNF, and apoptosis signaling pathways. (**f**) qPCR detection of gene expression (Tnf, Gadd45b, Atp5j2, and Nos2). (**g**) Western blot of the expression of proteins (Tnf, Gadd45b, Atp5j2, and Nos2) after different treatments and (**h**) corresponding quantitative analysis. Error bar represents ± S.D. (*n* = 3). **p < 0.01, ***p < 0.001

Inspired by the above exciting results, we conducted RNA expression sequencing (RNAseq) analysis to explore the biological mechanism of a-CoBiMn-LDH-PEG killing 4T1 cells. 4T1 cells treated with a-CoBiMn-LDH-PEG + H_2_O_2_ + US and PBS were labeled as experiment and control groups, respectively. Principal component analysis (PCA) and heat maps showed significant differences in transcriptomes between the control group and the experiment group (Fig. S18 and 5a). The volcano plot results showed that a total of 4902 genes were significantly differentially expressed, of which 2452 genes were up-regulated and 2450 genes were down-regulated (Fig. 5b). In view of this, gene ontology (GO) analysis was performed to reveal the therapeutic effects of a-CoBiMn-LDH-PEG on 4T1 cells. It was found that a-CoBiMn-LDH-PEG + H_2_O_2_ + US had a significant impact on the metabolic-related functions, cellular components, and biological processes of 4T1 cells (Fig. 5c). We also conducted Kyoto Encyclopedia of Genes and Genomes (KEGG) pathway enrichment analysis (Fig. 5d), which revealed significant changes in pathways related to oxidative phosphorylation, p53, TNF, apoptosis, and hypoxia after treatment with a-CoBiMn-LDH-PEG + H_2_O_2_ + US. Based on these results, we conducted heat map analysis of the corresponding signaling pathways (Fig. 5e), and the results showed that the treatment of a-CoBiMn-LDH-PEG + H_2_O_2_ + US significantly induced the up-regulation of apoptosis-related genes (such as Tnf, Gadd45b, and Gadd45g), and activated the p53 signaling pathway through ROS-mediated DNA damage, synergistically promoting cell apoptosis. In addition to the significant differential expression of p53 and apoptosis-related genes, the oxidative phosphorylation-related genes (Ndufa3, Cox7a2, Atp5j2) were down-regulated, indicating that a-CoBiMn-LDH-PEG + H_2_O_2_ could promote ROS production and inhibit oxidative phosphorylation in an ROS-dependent manner under US-assisted SDT treatment. Moreover, TNF signaling pathway-related genes such as Fos, Junb, and Cxcl2 were up-regulated, which increased the permeability of intracellular mitochondria and promotes the production of ROS. Furthermore, heat map analysis of HIF-1-related genes (Nos2 and Eno2) were down-regulated (Fig. S19), suggesting that a-CoBiMn-LDH-PEG + H_2_O_2_ under US irradiation could alleviate hypoxia. In addition, gene set enrichment analysis (GSEA) revealed significant positive enrichment scores for glucose catabolism, glycolysis, and apoptosis regulation (Fig. S20), demonstrating that a-CoBiMn-LDH-PEG + H_2_O_2_ + US could induce energy crisis and promote cell apoptosis.

To validate the differential expression of RNAseq genes, real-time fluorescence quantitative polymerase chain reaction (PCR) analysis was performed. As shown in Fig. 5f, apoptotic genes (Tnf, Gadd45b) were up-regulated, while oxidative phosphorylation gene (Atp5j2) and hypoxia-related gene (Nos2) were down-regulated, validating the reliability of the above RNAseq results. Western blotting analysis was further conducted to confirm the above results. In Fig. 5g and h, compared with PBS, US, and CoBiMn-LDH-PEG + US groups, the expression of apoptotic proteins (Tnf and Gadd45b) was higher in a-CoBiMn-LDH-PEG + US group, which was the highest in a-CoBiMn-LDH-PEG + H_2_O_2_ + US group. The expression of Atp5j2 and Nos2 proteins were significantly down-regulated after a-CoBiMn-LDH-PEG + H_2_O_2_ + US treatment, which was consistent with the results of PCR analysis. Taken together, these results indicated that a-CoBiMn-LDH-PEG + H_2_O_2_ + US played an important role in promoting cancer cell apoptosis and alleviating hypoxic microenvironment.

### *In vivo* SDT treatment

**Fig. 6:**
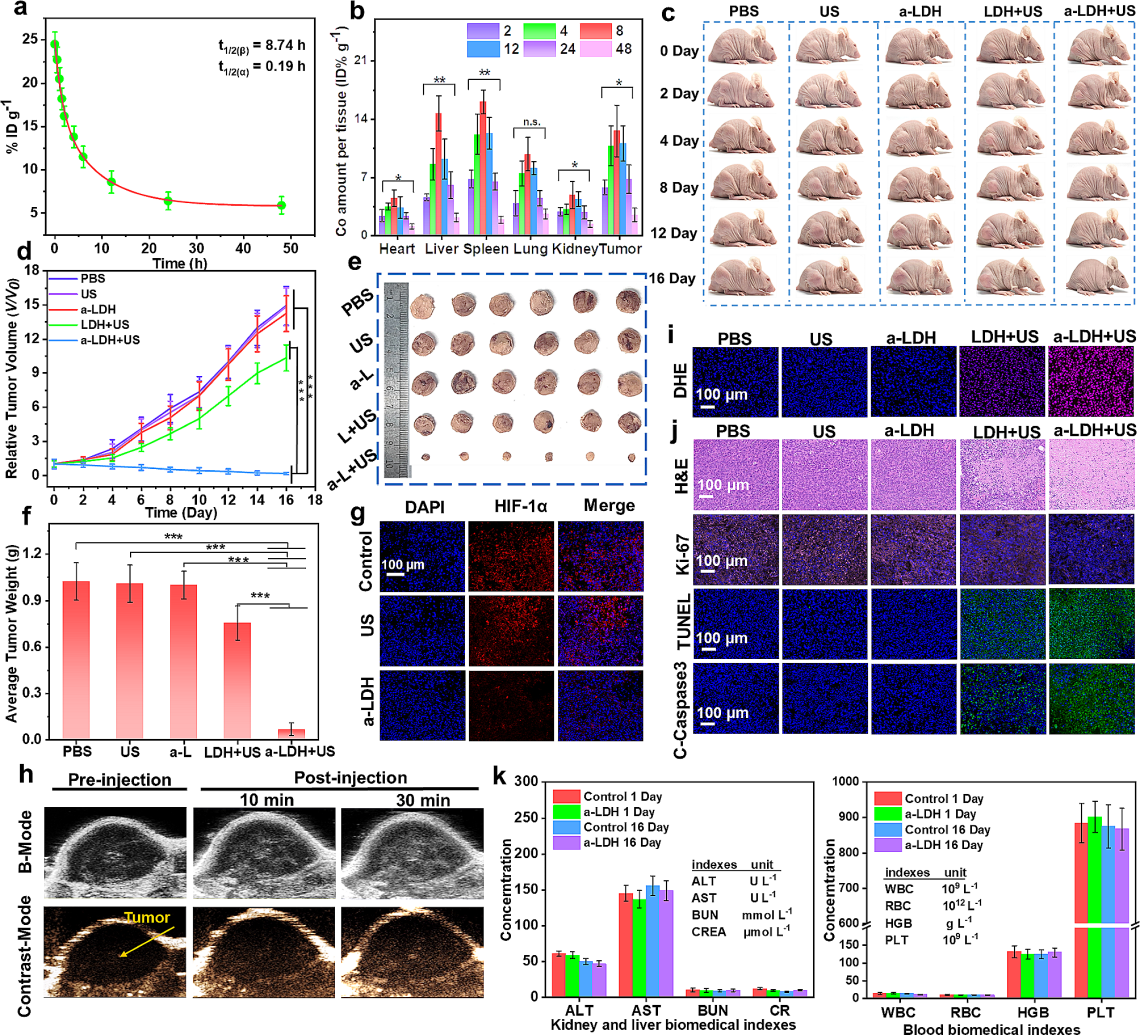
(**a**) Blood circulation time of a-CoBiMn-LDH-PEG in 4T1 tumor-bearing mice, quantified by determining the content of Co at each time point after injection. (**b**) Quantitative analysis of the biodistribution of a-CoBiMn-LDH-PEG in mice, also determined by measuring the Co concentration at various time points after injection. Error bar represents ± S.D. (*n* = 3). (**c**) Representative photos of 4T1 tumor-bearing mice given different treatments. (**d**) Tumor growth curves of 4T1 tumor-bearing mice after various treatments (PBS, US (40 kHz, 3 W cm^− 2^, 6 min), a-CoBiMn-LDH-PEG, CoBiMn-LDH-PEG + US, a-CoBiMn-LDH-PEG + US). Error bar represents ± S.D. (*n* = 6). (**e**) Representative photos of tumors on the 16th day after different treatments. (**f**) Average tumor weight of each group of mice after 16 days of treatment. Error bar represents ± S.D. (*n* = 6). (**g**) HIF-1α staining images of tumor sections in each group after 16 days. (**h**) In vivo US imaging. (**i**) DHE and (**j**) H&E, Ki-67, TUNEL, and C-Caspase3 staining analysis of tumor sections after 16 days of treatment. Each experiment was repeated three times. (**k**) Liver and kidney function indicators and blood cell count of mice on the 1st and 16th days after injection of PBS (control) and a-CoBiMn-LDH-PEG. Error bar represents ± S.D. (*n* = 3). *p < 0.05, **p < 0.01, ***p < 0.001

Motivated by the promising in vitro results, the therapeutic effect of a-CoBiMn-LDH-PEG was evaluated in vivo. The pharmacokinetics were first investigated. The blood circulation curve of the a-CoBiMn-LDH-PEG nanoparticles was fitted with quadratic exponents, and the calculated circulatory half-lives are 0.19 (t_1/2(α)_) and 8.74 h (t_1/2(β)_), respectively (Fig. 6a). Such a long blood circulation is conducive to the accumulation of a-CoBiMn-LDH-PEG at the tumor site. Subsequently, the biodistribution of a-CoBiMn-LDH-PEG was also studied. Hearts, livers, spleens, lungs, kidneys, and tumors were gathered at 2, 4, 8, 12, 24, and 48 h post-injection for inductively coupled plasma-atomic emission spectroscopy (ICP-AES) analysis. It was found that a-CoBiMn-LDH-PEG nanoparticles tended to accumulate in liver, spleen and tumor tissue, with the highest accumulation occurring at 8 h post-injection (Fig. 6b).

Subsequently, mice bearing 4T1 tumors were randomly divided into five groups (*n* = 6): (1) PBS, (2) US, (3) a-CoBiMn-LDH-PEG, (4) CoBiMn-LDH-PEG + US, (5) a-CoBiMn-LDH-PEG + US. Tumor size and bodyweight of the mice were recorded every two days for 16 days. Photographs of the mice at different time points and tumor growth measurements showed the significant tumor growth in the PBS, US irradiation and a-CoBiMn-LDH-PEG groups (Fig. 6c and d), whereas the tumor growth was slightly inhibited in mice injected with CoBiMn-LDH-PEG + US. In contrast, mice injected with a-CoBiMn-LDH-PEG showed complete regression of the tumor under US irradiation. The tumor tissues were gathered and weighed after 16 days of treatment. Digital images of the tumors (Fig. 6e) and the average tumor weight values (Fig. 6f) verified the significant inhibitory effect of a-CoBiMn-LDH-PEG plus US irradiation on tumor growth.

The O_2_-generating activity of a-CoBiMn-LDH-PEG nanoparticles was evaluated in vivo. Hypoxia-inducible factor-1α (HIF-1α) was used as a hypoxia-related marker to detect the O_2_ level in the tumor tissue. In Fig. 6g, obvious red fluorescence was observed in tumor tissues from the PBS- and US-treated mice, indicating the hypoxic state of the tumor. However, the red fluorescence in tumor tissues of mice injected with a-CoBiMn-LDH-PEG nanoparticles was markedly reduced, indicating that a-CoBiMn-LDH-PEG could relieve hypoxia levels by decomposing H_2_O_2_ to generate O_2_. In view of this, we further investigated the ability of a-CoBiMn-LDH-PEG nanoparticles for US imaging in vitro and in vivo. As shown in Fig. S21, a larger number of oxygen bubbles (white spots) were observed in the a-CoBiMn-LDH-PEG + H_2_O_2_ group compared with a-CoBiMn-LDH-PEG alone, especially at pH = 6.5, indicating the potential of a-CoBiMn-LDH-PEG for US imaging by generating O_2_. 4T1 tumor-bearing mice were thus injected with a-CoBiMn-LDH-PEG to conduct US imaging at different time points (see Fig. 6h). Compared with the situation before injection, the US signal contrast at the tumor site was significantly enhanced 30 min post-injection, proving that a-CoBiMn-LDH-PEG nanoparticles could not only effectively overcome tumor hypoxia, but also serve as a US imaging contrast agent [55–57].

Dihydroethidium (DHE) staining was performed on tumor tissues to detect ROS generation. As shown in Fig. 6i, the DHE fluorescence of the a-CoBiMn-LDH-PEG + US group was the strongest, suggesting the generation of a large amount of ROS and effective SDT performance. The in vivo therapeutic mechanism was further investigated through histological staining. Hematoxylin and eosin (H&E) staining images revealed that no significant damage was found in tumor tissues from the PBS, US and a-CoBiMn-LDH-PEG groups, while CoBiMn-LDH-PEG + US led to moderate cell apoptosis. In contrast, a-CoBiMn-LDH-PEG + US induced the most significant tumor cell apoptosis (Fig. 6j), demonstrating the most potent SDT effect of all the treatments. Ki-67 staining images (Fig. 6j) showed that a-CoBiMn-LDH-PEG markedly reduced the number of Ki-67 positive cells in tumor tissues under US irradiation, indicating its strong inhibitory effect on tumor proliferation. According to TUNEL and C-Caspase3 staining images (Fig. 6j), the a-CoBiMn-LDH-PEG + US group exhibited the strongest green fluorescence, confirming extensive cancer cell apoptosis.

In vivo biocompatibility of the a-CoBiMn-LDH-PEG nanoparticles was studied. As presented in Fig. S22, there was no significant change in the body weight of any group of mice during administration, suggesting the excellent biocompatibility of a-CoBiMn-LDH-PEG. To further evaluate the toxicity of a-CoBiMn-LDH-PEG, blood was collected on day 1 and day 16 after intravenous injection, and routine blood examinations and blood biochemical analysis were performed. It was found that blood parameters and liver/kidney function markers were not significantly different from those of the PBS group (Fig. 6k), indicating negligible blood toxicity of a-CoBiMn-LDH-PEG. H&E staining images of the heart, kidney, liver, lung, and spleen after the end of the treatment period showed that there was no obvious physiological abnormality or difference between the a-CoBiMn-LDH-PEG and PBS groups (Fig. S23), again indicating low toxicity. The survival time of mice in each group was also recorded. In Fig. S24, it can be seen that the a-CoBiMn-LDH-PEG + US treated mice remained healthy for 60 days after treatment, without tumor recurrence. In contrast, the other groups of mice died to various extents over this period of time, confirming the therapeutic efficiency of a-CoBiMn-LDH-PEG nanoparticles under US irradiation. The Co content in urine and feces was determined by ICP-AES to explore the metabolism of a-CoBiMn-LDH-PEG nanoparticles in vivo. As shown in the Fig. S25, a high concentration of Co was detected at 8 h post-injection and this then gradually decreased, demonstrating that a-CoBiMn-LDH-PEG nanoparticles could be metabolized effectively and excreted in the feces and urine. In summary, the above results prove that a-CoBiMn-LDH-PEG nanoparticles possess good biocompatibility, potentiating its further application as a highly active sonosensitizer for SDT.

## Conclusion

In this work, a new LDH-based sonosensitizer with high ROS generation activity, TME remodeling ability and multifunctionality has been fabricated for highly efficient US-imaging-guided SDT. A simple acid etching treatment can adjust the electronic structure and properties of CoBiMn-LDH nanoparticles to obtain defect-rich amorphous a-CoBiMn-LDH nanoparticles, and significantly strengthen the system’s ROS generation performance under US irradiation (to ~ 3.3 times that of the crystalline CoBiMn-LDH nanoparticles and ~ 8.2 times that of the commercial TiO_2_ sonosensitizer). Mn^4+^ present in the system enables the a-CoBiMn-LDH nanoparticles to regulate the TME through two processes: (a) decomposing H_2_O_2_ into O_2_ to relieve tumor hypoxia and mediate US imaging; (b) consuming GSH to reduce the clearance of ROS. After PEG modification, a-CoBiMn-LDH-PEG nanoparticles are found to effectively kill cancer cells and eliminate tumors under US irradiation, as evidenced by in vitro and in vivo assays. Moreover, biological mechanism analysis further revealed that a-CoBiMn-LDH-PEG nanoparticles could activate p53, apoptosis, and oxidative phosphorylation-related signaling pathways under US irradiation to exert therapeutic effect. This work provides a promising paradigm for the preparation of LDH-based sonosensitizers for imaging-guided SDT, demonstrating enormous potential for clinical practice.

## Materials and methods

### Preparation of CoBiMn-LDH nanoparticles

Solution A (50 mL) was composed of Bi(NO_3_)_3_·5H_2_O (0.0001 mol), Co(NO_3_)_2_·6H_2_O (0.0015 mol) and Mn(NO_3_)_2_·2H_2_O (0.0004 mol). NaOH (0.0125 mol) was dissolved in 50 mL deoxygenated water as solution B. 10 mL of solution A and 9 mL of solution B were simultaneously dropped into a three-nozzle flask containing 10 mL of deoxygenated water. The resultant sediments were magnetically stirred at 1000 RPM for 30 min in a nitrogen (N_2_) atmosphere at 90 °C. After cooling to room temperature, the reaction gel was centrifuged at 8500 RPM for 5 min. The final CoBiMn-LDH nanoparticles were obtained after washing the sediments three times with deoxygenated water. The deoxygenated water used in the experiment was prepared by boiling deionized water under a N_2_ atmosphere.

### Preparation of a-CoBiMn-LDH nanoparticles

The prepared CoBiMn-LDH (15.6 mg) precursor was dispersed in a buffer solution (pH = 4.0) (2.5 mL) and stirred at room temperature for 10 h. After centrifugation at 8500 RPM for 5 min and washing with deoxygenated water three times, the final a-CoBiMn-LDH nanoparticles were obtained and stored in deoxygenated water for further use.

### Preparation of a-CoBiMn-LDH-PEG nanoparticles

0.03 mol of PEG (100 mg) was added to a-CoBiMn-LDH (10 mg, 1 mg mL^− 1^) suspension and stirred at room temperature for 24 h. After centrifugation at 8500 RPM for 5 min and washing three times with deoxygenated water, the final a-CoBiMn-LDH-PEG nanoparticles were obtained.

### Detection of ^1^O_2_ by SOSG

CoBiMn-LDH, a-CoBiMn-LDH, and commercial TiO_2_ (0.2 mL, 1 mg mL^− 1^) were mixed with SOSG (400 µL, 0.03 M), respectively, and then added into 1400 µL of a buffer solution (pH = 6.5). ^1^O_2_ generation was detected by recording the fluorescence intensity of SOSG at 526 nm under US irradiation (40 kHz, 3 W cm^− 2^).

### Detection of ^1^O_2_ by DPBF

Four experimental groups were established: (1) CoBiMn-LDH (200 µL,1 mg mL^− 1^), (2) a-CoBiMn-LDH (200 µL,1 mg mL^− 1^), (3) TiO_2_ (200 µL,1 mg mL^− 1^), (4) a-CoBiMn-LDH (200 µL,1 mg mL^− 1^) + H_2_O_2_ (200 µL, 1 mM). These suspensions were individually added into DPBF solution (50 µL, 1 mg mL^− 1^) and mixed evenly, followed by the addition of buffer solution (pH = 6.5) to reach a total volume of 2 mL. The mixtures were then exposed to US irradiation (40 kHz, 3 W cm^− 2^) for a total of 6 min. The UV absorption spectra of DPBF were recorded every minute to indirectly detect the generation of ^1^O_2_.

### Detection of ^1^O_2_ by ESR

The generation of ^1^O_2_ was further confirmed by ESR spectroscopy with TEMP as a probe. Briefly, TEMP solution (200 µL, 0.03 M) was mixed with the following seven groups: (1) control, (2) US (40 kHz, 3 W cm^− 2^), (3) a-CoBiMn-LDH (200 µL, 1 mg mL^− 1^), (4) TiO_2_ (200 µL, 1 mg mL^− 1^) + US, (5) CoBiMn-LDH (200 µL, 1 mg mL^− 1^) + US, (6) a-CoBiMn-LDH (200 µL, 1 mg mL^− 1^) + US, (7) a-CoBiMn-LDH (200 µL, 1 mg mL^− 1^) + H_2_O_2_ (200 µL, 1 mM) + US. This was followed by the addition of buffer solution (pH = 6.5) to reach a total volume of 2 mL. After US irradiation for 6 min, the ^1^O_2_ generation of mixture was detected by ESR.

### O_2_ generation assessment

H_2_O_2_ (0.1 mM) and a-CoBiMn-LDH (100 µg mL^− 1^) were mixed in buffer solutions at pH = 6.5 and 7.4 to reach a total volume of 2 mL, respectively. a-CoBiMn-LDH alone was used as a control. The generation of O_2_ was measured within 10 min using a portable dissolved oxygen meter.

### Depletion of GSH

GSH solution (0.2 mM) was incubated with various concentrations (0, 25, 50, 75, 100 µg mL^− 1^) of a-CoBiMn-LDH nanoparticles (total volume of 2 mL) at room temperature for 4 h, followed by the addition of DTNB (200 µL, 2.5 mg mL^− 1^). The GSH content was then detected with a UV-vis spectrophotometer.

### Cell culture

4T1, Hela and HepG2 cell lines were purchased from the Institute of Basic Medicine Chinese Academy of Medical Sciences (Beijing, China). All cells were cultured with DMEM medium containing 1% antibiotic solution (penicillin/streptomycin) and 10% FBS in 25 cm^2^ cell culture flasks in a 37 ℃ and 5% CO_2_ atmosphere. The cells were separated from the flasks by adding 2 mL 0.25% trypsin, and then incubated with fresh DMEM medium for subsequent experiments.

**In vitro MTT assay**

The MTT assay was used to assess the in vitro biocompatibility and cytotoxicity. 4T1, Hela, or HepG2 cells were first inoculated into 96-well plates (1 × 10^4^ per well). Then, the cells were incubated in DMEM medium (pH = 6.5, 200 µL/well) containing different concentrations of a-CoBiMn-LDH-PEG (10, 20, 50, 100 and 200 µg mL^− 1^) for 24 h. After removing the medium and washing with PBS three times, 0.5 mg mL^− 1^ MTT (200 µL) was added for 4 h incubation. The absorbance of the reaction product (formazan) at 490 nm was determined with an automatic microplate reader (Synergy H1, BioTek Instruments, Inc). For the cytotoxicity test, 4T1 cells were separated into six groups as follows: (1) control, (2) US (40 kHz, 3 W cm^− 2^), (3) a-CoBiMn-LDH-PEG (100 µg mL^− 1^), (4) CoBiMn-LDH-PEG (100 µg mL^− 1^) + US, (5) a-CoBiMn-LDH-PEG (100 µg mL^− 1^) + US, (6) a-CoBiMn-LDH-PEG (100 µg mL^− 1^) + H_2_O_2_ (0.1 mM) + US. US irradiation was performed at 8 h post-administration. After 24 h of incubation, the DMEM medium (pH = 6.5) was discarded and the cells were rinsed with PBS three times. Then, MTT (200 µL, 0.5 mg mL^− 1^) was added to incubate for 4 h. Finally, the cell viability was evaluated by detecting the absorbance of the reaction product (formazan) at 490 nm.

### Calcein-AM/PI staining

To confirm the MTT results, Calcein-AM/PI double staining assay was performed. 4T1 cells were seeded into 6-well plates (1 × 10^5^ per well, 2 mL) for 24 h and then divided into six groups: (1) control, (2) US (40 kHz, 3 W cm^− 2^), (3) a-CoBiMn-LDH-PEG (100 µg mL^− 1^), (4) CoBiMn-LDH-PEG (100 µg mL^− 1^) + US, (5) a-CoBiMn-LDH-PEG (100 µg mL^− 1^) + US, (6) a-CoBiMn-LDH-PEG (100 µg mL^− 1^) + H_2_O_2_ (0.1 mM) + US (performed at 8 h post-administration). After 24 h of incubation, the DMEM medium (pH = 6.5) was discarded and the cells were rinsed with PBS three times. Subsequently, the 4T1 cells were stained with 2 mL DMEM of 0.01 mg mL^− 1^ Calcein-AM and 0.015 mg mL^− 1^ PI for 20 min. Finally, the cells were rinsed with PBS and then imaged with a Leica microscope (DMi8, Germany).

### Apoptosis and necrosis assay

4T1 cells were seeded into 6-well plates (1 × 10^5^ per well, 2 mL) for 24 h and then the medium was replaced with fresh DMEM (pH = 6.5) containing 100 µg mL^− 1^ CoBiMn-LDH-PEG, a-CoBiMn-LDH-PEG and a-CoBiMn-LDH-PEG + H_2_O_2_ (0.1 mM). After incubation for 8 h, the cells were in some cases subject to US irradiation (40 kHz, 3 W cm^− 2^, 6 min). After continued culture for 16 h, live and dead cells were collected and stained with Annexin V-FITC (10 µg mL^− 1^) and PI (5 µg mL^− 1^) for 30 min. Finally, the apoptosis was studied by flow cytometry.

### Generation of intracellular ROS

The ROS levels of 4T1 cells were detected by DCFH-DA staining. Briefly, 4T1 cells (5 × 10^4^ cells mL^− 1^, 2 mL) were incubated in 6-well plates for 24 h and then the media replaced with fresh DMEM (pH = 6.5) media containing 100 µg mL^− 1^ CoBiMn-LDH-PEG, a-CoBiMn-LDH-PEG and a-CoBiMn-LDH-PEG + H_2_O_2_ (0.1 mM). After 8 h of incubation, US irradiation (40 kHz, 3 W cm^− 2^) was applied in some cases, for 6 min. Afterwards, all treated cells were stained with DCFH-DA (30 µg mL^− 1^) for 20 min. Finally, fluorescence images were collected with a Leica microscope (DMi8, Germany).

### Mitochondrial membrane potential and lysosome disruption assays

4T1 cells were separated into six groups as follows and incubated for 24 h: (1) control, (2) US (40 kHz, 3 W cm^− 2^), (3) a-CoBiMn-LDH-PEG (100 µg mL^− 1^), (4) CoBiMn-LDH-PEG (100 µg mL^− 1^) + US, (5) a-CoBiMn-LDH-PEG (100 µg mL^− 1^) + US, (6) a-CoBiMn-LDH-PEG (100 µg mL^− 1^) + H_2_O_2_ (0.1 mM) + US. US irradiation was performed at 8 h post-administration. After staining with JC-1 (2 mL, 10 µg mL^− 1^) for 30 min, the fluorescence images of cells were collected by CLSM (Leica DM6000M, Germany). Lysosomal destruction assay was conducted by similar procedures except that JC-1 was replaced by LysoTracker Green (2 mL, 10 µg mL^− 1^).

#### Hypoxia staining

O_2_ generation was measured using [Ru(dpp)_3_]Cl_2_ probe. Briefly, 4T1 cells were inoculated in 6-well culture plates and cultured at 37 ℃ and 5% CO_2_ for 24 h. Then, 4T1 cells were divided into three groups: (1) control, (2) a-CoBiMn-LDH-PEG (100 µg mL^− 1^), (3) a-CoBiMn-LDH-PEG + H_2_O_2_ (0.1 mM). Subsequently, all treated cells were incubated with [Ru(dpp)_3_]Cl_2_ (2 mL, 10 µM) for 4 h under anoxic environment. Finally, the fluorescence images of cells were captured by Leica fluorescence microscope (DMi8, Germany).

### Hemolysis evaluation

Red blood cells were collected from mice by centrifuging whole blood at 1500 RPM for 15 min, and then washed with PBS five times to prepare a red blood cell suspension. 200 µL of red blood cell suspension was added to a-CoBiMn-LDH-PEG suspensions at different concentrations (12.5 to 200 µg mL^− 1^) for 4 h. H_2_O was used as a positive control while PBS was a negative control. Subsequently, the supernatant was centrifuged at 10,000 RPM for 5 min, and the absorbance at 570 nm was determined by ultraviolet spectroscopy. The hemolysis rate was calculated as:

Hemolysis rate (%) = (sample absorbance - negative control absorbance)/(positive control absorbance - negative control absorbance) × 100%.

#### Animal experiments

Female Balb/c nude mice aged 4 ~ 6 weeks (weight 18 ~ 22 g) were acquired from Beijing Vital River Laboratory Animal Technology Co., Ltd. 4T1 cells (100 µL, 1 × 10^7^) suspended in PBS were subcutaneously inoculated into the right hind leg of the mice to establish a xenograft model. When the average volume of the tumor reached 80 mm^3^, follow-up experiments could be performed. The tumor volume could be calculated by the formula: tumor volume = (tumor width × tumor width × tumor length)/2.

#### In vivo pharmacokinetic evaluation

To investigate the pharmacokinetic behavior of a-CoBiMn-LDH-PEG, mice bearing 4T1 tumors (*n* = 3) were injected intravenously with a-CoBiMn-LDH-PEG (0.2 mL, 1 mg mL^− 1^). Subsequently, blood samples were gathered at specific time points (0, 0.25, 0.5, 1, 2, 4, 8, 12, 24, 48 h). Finally, the Co content in the blood was determined using ICP-AES.

#### In vivo biodistribution assay

To study the biodistribution of a-CoBiMn-LDH-PEG, mice bearing 4T1 tumors (*n* = 3) were injected intravenously with a-CoBiMn-LDH-PEG (0.2 mL, 1 mg mL^− 1^). Then, the animals were sacrificed at specific time points (2, 4, 8, 12, 24, and 48 h) and the hearts, livers, spleens, lungs, kidneys, and tumors were gathered. After dissolution with nitric acid, the content of Co in these tissues was determined by ICP-AES.

#### In vivo SDT therapy

Mice bearing 4T1 tumors were randomly divided into five groups (*n* = 6): (1) PBS (200 µL), (2) US (200 µL PBS), (3) a-CoBiMn LDH-PEG (200 µL, 10 mg kg^− 1^), (4) CoBiMn-LDH-PEG (200 µL, 10 mg kg^− 1^) + US, (5) a-CoBiMn-LDH-PEG (200 µL, 10 mg kg^− 1^) + US. Mice in groups (2), (4) and (5) were irradiated with US (40 kHz, 3 W cm^− 2^) for 6 min at 8 h post-injection. The tumor size and body weight of the mice were monitored until the 16th day.

#### In vitro and in vivo US imaging

Small animal ultrasound imaging (VisualSonics Vevo2100) was used for in vitro and in vivo US imaging. First, a-CoBiMn-LDH-PEG (100 µg mL^− 1^) and H_2_O_2_ (1 mM) were mixed in buffers at pH = 6.5 and 7.4 (2 mL), respectively. a-CoBiMn-LDH-PEG (100 µg mL^− 1^) alone and H_2_O_2_ (1 mM) alone were used as control groups. Subsequently, an ultrasound scanning probe was used to scan and images were recorded. For in vivo US imaging, a-CoBiMn-LDH-PEG (200 µL, 10 mg kg^− 1^) was injected into mice bearing 4T1 tumors, and images were recorded at specific time points (0, 10, and 30 min). All measurements were taken at room temperature.

#### Pathological investigation

After 16 days of treatment, the above five groups of mice were sacrificed. The major organs (heart, liver, spleen, lung, kidney) and tumors were collected and soaked in a 4% paraformaldehyde solution before embedding them with paraffin blocks. All tissue sections were stained with hematoxylin and eosin (H&E) for histological damage analysis. Tumor sections were also stained with Ki-67, TUNEL, and C-Caspase3 to detect apoptosis. Staining was performed following the manufacturer instructions.

### Electronic supplementary material

Below is the link to the electronic supplementary material.

Supplementary Material 1

## Data Availability

Data is provided within the manuscript or supplementary information files.

## References

[1] Wang X, Zhong X, Gong F (2020). Newly developed strategies for improving sonodynamic therapy. Mater Horiz.

[2] Lin X, Song J, Chen X (2020). Ultrasound-activated sensitizers and applications. Angew Chem Int Ed.

[3] Zhang T, Sun Y, Cao J (2021). Intrinsic nucleus-targeted ultra-small metal-organic framework for the type I sonodynamic treatment of orthotopic pancreatic carcinoma. J Nanobiotechnol.

[4] Cao Y, Wang X, Song W (2023). Defect-Engineering Bismuth-based homologous Schottky Heterojunction for metabolic regulation-augmented Sonodynamic Tumor Therapy. Adv Funct Mater.

[5] Sun L, Cao Y, Lu Z (2022). A hypoxia-irrelevant Fe-doped multivalent manganese oxide sonosensitizer via a vacancy engineering strategy for enhanced sonodynamic therapy. Nano Today.

[6] Qian X, Zheng Y, Chen Y (2016). Micro/Nanoparticle-Augmented Sonodynamic Therapy (SDT): breaking the depth shallow of Photoactivation. Adv Mater.

[7] Wang J, Huang J, Zhou W (2021). Hypoxia modulation by dual-drug nanoparticles for enhanced synergistic sonodynamic and starvation therapy. J Nanobiotechnol.

[8] Gong F, Cheng L, Yang N (2019). Ultrasmall Oxygen-Deficient Bimetallic Oxide MnWO_X_ nanoparticles for Depletion of endogenous GSH and enhanced Sonodynamic Cancer Therapy. Adv Mater.

[9] Zhang M, Yang D, Dong C (2022). Two-dimensional MXene-Originated in situ Nanosonosensitizer Generation for Augmented and Synergistic Sonodynamic Tumor Nanotherapy. ACS Nano.

[10] Chen H, He X, Zhou Z (2022). Metallic phase enabling MoS_2_ nanosheets as an efficient sonosensitizer for photothermal-enhanced sonodynamic antibacterial therapy. J Nanobiotechnol.

[11] Wang X, Zhong X, Bai L (2020). Ultrafine Titanium Monoxide (TiO_1 + x_) nanorods for enhanced Sonodynamic Therapy. J Am Chem Soc.

[12] Chen W, Liu C, Ji X (2021). Stanene-based nanosheets for β-Elemene delivery and ultrasound-mediated Combination Cancer Therapy. Angew Chem Int Ed Engl.

[13] Lin X, Liu S, Zhang X (2020). An Ultrasound activated vesicle of Janus Au-MnO nanoparticles for promoted Tumor Penetration and Sono-Chemodynamic Therapy of Orthotopic Liver Cancer. Angew Chem Int Ed Engl.

[14] Bai S, Yang N, Wang X (2020). Ultrasmall Iron-Doped Titanium Oxide nanodots for enhanced Sonodynamic and Chemodynamic Cancer Therapy. ACS Nano.

[15] Liang S, Xiao X, Bai L (2021). Conferring Ti-Based MOFs with defects for enhanced Sonodynamic Cancer Therapy. Adv Mater.

[16] Yan S, Lu M, Ding X (2016). HematoPorphyrin Monomethyl Ether Polymer contrast agent for ultrasound/photoacoustic dual-modality imaging-guided synergistic high intensity focused ultrasound (HIFU) therapy. Sci Rep.

[17] Zou W, Hao J, Wu J (2022). Correction: biodegradable reduce expenditure bioreactor for augmented sonodynamic therapy via regulating tumor hypoxia and inducing pro–death autophagy. J Nanobiotechnol.

[18] Tan X, Huang J, Wang Y (2021). Transformable nanosensitizer with Tumor Microenvironment-activated sonodynamic process and calcium release for enhanced Cancer Immunotherapy. Angew Chem Int Ed Engl.

[19] Guan X, Yin HH, Xu XH (2020). Tumor Metabolism-Engineered Composite Nanoplatforms Potentiate Sonodynamic Therapy via reshaping Tumor Microenvironment and Facilitating Electron-Hole pairs’ separation. Adv Funct Mater.

[20] Wu T, Liu Y, Cao Y (2022). Engineering Macrophage Exosome disguised biodegradable nanoplatform for enhanced Sonodynamic Therapy of Glioblastoma. Adv Mater.

[21] Wu Q, Zhang F, Pan X (2021). Surface wettability of nanoparticle modulated Sonothrombolysis. Adv Mater.

[22] Zhang T, Zheng Q, Fu Y (2021). α-Fe_2_O_3_@Pt heterostructure particles to enable sonodynamic therapy with self-supplied O_2_ and imaging-guidance. J Nanobiotechnol.

[23] Ma A, Chen H, Cui Y (2019). Metalloporphyrin Complex-based nanosonosensitizers for deep-tissue Tumor Theranostics by Noninvasive Sonodynamic Therapy. Small.

[24] Liu Z, Robinson JT, Sun X (2008). PEGylated nanographene oxide for delivery of water-insoluble cancer drugs. J Am Chem Soc.

[25] Chen Y, Ye D, Wu M (2014). Break-up of two-dimensional MnO_2_ nanosheets promotes ultrasensitive pH-triggered theranostics of cancer. Adv Mater.

[26] Hu T, Gu Z, Williams GR (2022). Layered double hydroxide-based nanomaterials for biomedical applications. Chem Soc Rev.

[27] Gu Z, Atherton JJ, Xu ZP (2015). Hierarchical layered double hydroxide nanocomposites: structure, synthesis and applications. Chem Commun.

[28] Wang G, Lv Z, Wang T (2022). Surface functionalization of Hydroxyapatite scaffolds with MgAlEu-LDH nanosheets for high-performance bone regeneration. Adv Sci.

[29] Zhu Y, Wang Y, Williams GR (2020). Multicomponent Transition Metal Dichalcogenide nanosheets for imaging-guided Photothermal and Chemodynamic Therapy. Adv Sci.

[30] Mei X, Ma J, Bai X (2018). A bottom-up synthesis of rare-earth-hydrotalcite monolayer nanosheets toward multimode imaging and synergetic therapy. Chem Sci.

[31] Gao R, Mei X, Yan D (2018). Nano-Photosensitizer based on layered double hydroxide and isophthalic acid for singlet oxygenation and photodynamic therapy. Nat Commun.

[32] Hu T, Shen W, Meng F (2023). Boosting the Sonodynamic Cancer Therapy performance of 2D layered double hydroxide nanosheet-based Sonosensitizers Via Crystalline-to-Amorphous Phase Transformation. Adv Mater.

[33] Chen M, Dong C, Shi S (2022). An overview of recent advancements on manganese-based nanostructures and their application for ROS-mediated tumor therapy. ACS Mater Lett.

[34] Liu J, Chen C, Chen H (2022). Brain glucose activated MRI contrast Agent for early diagnosis of Alzheimer’s Disease. Anal Chem.

[35] Wu F, Du Y, Yang J (2022). Peroxidase-like active nanomedicine with Dual Glutathione Depletion Property to Restore Oxaliplatin Chemosensitivity and promote programmed cell death. ACS Nano.

[36] Zhao Y, Zhao Y, Waterhouse GIN (2017). Layered-Double-Hydroxide Nanosheets as efficient visible-light-driven photocatalysts for Dinitrogen fixation. Adv Mater.

[37] Li B, Gu Z, Kurniawan N (2017). Manganese-based layered double hydroxide nanoparticles as a T1 -MRI contrast Agent with Ultrasensitive pH response and high relaxivity. Adv Mater.

[38] Wang S, Yang S, Cui Z (2023). Chen, In-situ activation of CuAl-LDH nanosheets to catalyze Bioorthogonal Chemistry in vivo in Tumor Microenvironment for Precise Chemotherapy and Chemodynamic Therapy. Chem Eng J.

[39] Shen W, Hu T, Liu X (2022). Defect engineering of layered double hydroxide nanosheets as inorganic photosensitizers for NIR-III photodynamic cancer therapy. Nat Commun.

[40] Yu B, Wang W, Sun W (2021). Defect Engineering enables synergistic action of enzyme-mimicking active centers for High-Efficiency Tumor Therapy. J Am Chem Soc.

[41] Yuan X, Wang L, Hu M (2021). Oxygen Vacancy-Driven Reversible Free Radical Catalysis for Environment-Adaptive Cancer Chemodynamic Therapy. Angew Chem Int Ed Engl.

[42] Zhao Z, Wang W, Li C (2019). Reactive oxygen species-Activatable liposomes regulating hypoxic Tumor Microenvironment for Synergistic Photo/Chemodynamic therapies. Adv Funct Mater.

[43] Jia Q, Ge J, Liu W (2018). A Magnetofluorescent Carbon Dot Assembly as an acidic H_2_O_2_-Driven oxygenerator to regulate Tumor Hypoxia for Simultaneous Bimodal Imaging and enhanced photodynamic therapy. Adv Mater.

[44] Zhong X, Wang X, Zhan G (2019). NaCeF4: Gd, Tb Scintillator as an X-ray responsive photosensitizer for Multimodal Imaging-guided synchronous Radio/Radiodynamic therapy. Nano Lett.

[45] Jin S, Shao W, Luo X (2022). Spatial Band separation in a Surface Doped Heterolayered structure for realizing efficient Singlet Oxygen Generation. Adv Mater.

[46] Ji X, Ge L, Liu C (2021). Capturing functional two-dimensional nanosheets from sandwich-structure vermiculite for cancer theranostics. Nat Commun.

[47] Zhang Y, Wei S (2019). Mg-Co-Al-LDH nanoparticles with attractive electrochemical performance for supercapacitor. J Nanopart Res.

[48] Li L, Yang Y, Wang Y (2020). Electrochemical activity of layered double hydroxides supported nano pt clusters toward methanol oxidation reaction in alkaline solutions. J Mater Res Technol.

[49] Chen D, Yu Q, Huang X (2020). A highly-efficient type I photosensitizer with robust vascular-disruption activity for hypoxic-and-metastatic Tumor Specific Photodynamic Therapy. Small.

[50] Liu T, Wang C, Gu X (2014). Drug delivery with PEGylated MoS_2_ Nano-sheets for combined Photothermal and Chemotherapy of Cancer. Adv Mater.

[51] Yang J, Xie R, Feng L (2019). Hyperthermia and Controllable Free Radical Co-enhanced synergistic therapy in Hypoxia enabled by Near-Infrared-II light irradiation. ACS Nano.

[52] Liu G, Zou J, Tang Q (2017). Surface modified Ti_3_C_2_ MXene nanosheets for Tumor Targeting Photothermal/Photodynamic/Chemo synergistic therapy. ACS Appl Mater Interfaces.

[53] Zhang C, Chen WH, Liu LH (2017). An O_2_ self-supplementing and reactive-oxygen-species-circulating amplified Nanoplatform via H_2_O/H_2_O_2_ splitting for Tumor Imaging and photodynamic therapy. Adv Funct Mater.

[54] Hu T, Yan L, Wang Z (2021). A pH-responsive ultrathin Cu-based nanoplatform for specific photothermal and chemodynamic synergistic therapy. Chem Sci.

[55] Pan X, Wang W, Huang Z (2020). MOF-Derived double-layer Hollow nanoparticles with Oxygen Generation ability for Multimodal Imaging-guided Sonodynamic Therapy. Angew Chem Int Ed Engl.

[56] Sun L, Zhang J, Xu M (2022). Ultrasound Microbubbles mediated sonosensitizer and antibody co-delivery for highly efficient synergistic therapy on HER2-Positive gastric Cancer. ACS Appl Mater Interfaces.

[57] Jia X, Cai X, Chen Y (2015). Perfluoropentane-encapsulated Hollow Mesoporous prussian blue nanocubes for activated Ultrasound Imaging and Photothermal Therapy of Cancer. ACS Appl Mater Interfaces.

