# Supplementary material for: Boosting the sonodynamic performance of CoBiMn-layered double hydroxide nanoparticles via tumor microenvironment regulation for ultrasound imaging-guided sonodynamic therapy
Source: J Nanobiotechnology. 2024 Jun 8;22:317. doi: 10.1186/s12951-024-02591-5 (PMC11161954; doi:10.1186/s12951-024-02591-5)
Supplement: Supplementary file 1 — Supplementary Material 1 [file 12951_2024_2591_MOESM1_ESM.docx]

**Supporting information**

**Boosting the Sonodynamic Performance of CoBiMn-Layered Double Hydroxide Nanoparticles via Tumor Microenvironment Regulation for Ultrasound Imaging-Guided Sonodynamic Therapy**

Shuqing Yang^a,#^, Tingting Hu^b,#^, Gareth R. Williams^c^, Yu Yang^a^, Susu Zhang^d^, Jiayi Shen^d^, Minjiang Chen^d,^*, Ruizheng Liang^a,e,^*, Lingchun Lyu^d,^*

^#^ These authors contributed equally: Shuqing Yang and Tingting Hu.

^a^ State Key Laboratory of Chemical Resource Engineering, Beijing Advanced Innovation Center for Soft Matter Science and Engineering, Beijing University of Chemical Technology, Beijing, 100029, P. R. China

^b^ Department Electrical and Electronic Engineering, The University of Hong Kong, Pokfulam Road, Hong Kong SAR 999077, P. R. China

^c^ UCL School of Pharmacy, University College London, 29-39 Brunswick Square, London WC1N 1AX, UK

^d^ Lishui Central Hospital and the Fifth Affiliated Hospital of Wenzhou Medical University, Lishui, 323000, P. R. China

^e^ Quzhou Institute for Innovation in Resource Chemical Engineering, Quzhou 324000, P. R. China

E-mail: [minjiangchen@wmu.edu.cn](mailto:minjiangchen@wmu.edu.cn) (M. Chen), [liangrz@mail.buct.edu.cn](mailto:liangrz@mail.buct.edu.cn) (R. Liang), [lvlingchun@medmail.com.cn](mailto:lvlingchun@medmail.com.cn) (L. Lyu)

**Materials**

Nitric acid (HNO_3_), cobalt nitrate hexahydrate (Co(NO_3_)_2_·6H_2_O, >99.0%), bismuth nitrate pentahydrate (Bi(NO_3_)_3_·5H_2_O, >99.0%), manganese nitrate dihydrate (Mn(NO_3_)_2_·2H_2_O, >99.0%), sodium hydroxide (NaOH, >98.0%), polyethylene glycoi 2000 (PEG-2000, HO(CH_2_CH_2_O)nH), H_2_O_2_ 30%), GSH (>98.0%), DPBF, DTNB, SOSG, TEMP, DHR 123, [Ru(dpp)_3_]Cl_2_ (luminescent oxygen sensor), and phosphate buffered solution (PBS) were bought from Sigma-Aldrich and Fisher (USA). DMEM, ciprofloxacin (CPFX), 0.25% trypsin-EDTA, and fetal bovine serum (FBS) were acquired from Gibco (Invitrogen, Carlsbad, CA). Penicillin/streptomycin and MTT were acquired from Macklin (Shanghai, China). Calcein-AM/PI, DCFH-DA, LysoTracker Green, JC-1, and Annexin V-FITC/PI apoptosis detection kit were obtained from Beyotime Biotechnology Co., Ltd. The above chemicals have not been further purified before use.

**Characterizations**

XRD patterns were characterized on Shimadzu XRD-6000 diffractometer with a Cu Kα radiation source (λ= 0.15406 nm). A HT7700-MS2 transmission electron microscope (HITACHI, Tokyo, Japan) with an accelerating voltage of 100 kV was used to collect TEM images. The thickness of the sample was determined by AFM (MultiMode 8, Bruker, USA) in tapping mode. XPS analysis was conducted on X-ray photoelectron spectrometer (Escalab 250Xi, Thermo Scientific, USA). ESR spectra were obtained with the aid of a EMX1598 spectrometer (Bruker, USA). The hydrodynamic size and zeta potential were measured by a Zetasizer instrument (Malvern, UK). The concentration of metal elements was detected by ICP-AES (Shimadzu ICPS-7500, Japan). Fluorescence intensity of SOSG was measured using a Hitachi F-7000 fluorescence spectrometer, and UV absorption spectra of DPBF using a UV spectrophotometer (Shimadzu UV-2600, Japan). Staining images of cells were collected on inverted fluorescence microscope (Leica DMi8, Germany) and confocal laser scanning microscope (CLSM, Leica DM6000M, Germany). Cell apoptosis was analyzed by flow cytometry (MoFlo XDP, Beckman Coulter, USA).

**Statistical analysis**

All data are expressed as mean ± standard deviation (s.d). One-way ANOVA was applied to analyze the statistical significance of the data (* p<0.05, ** p<0.01, *** p<0.001).


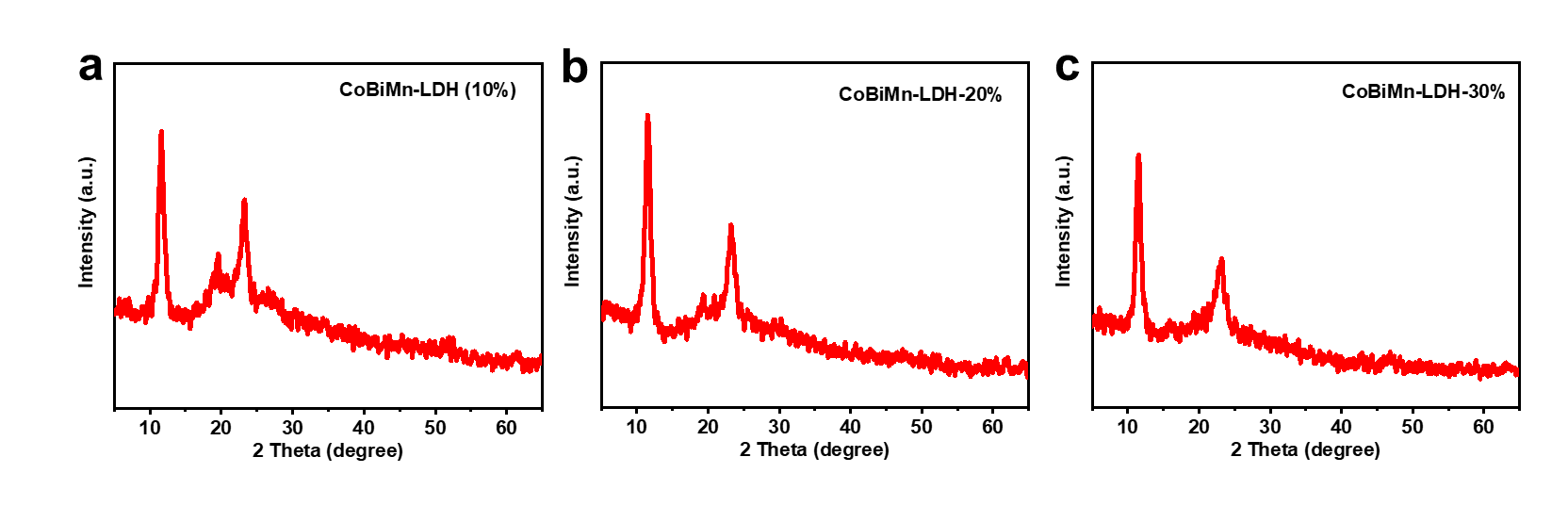


**Fig. S1** XRD patterns of CoBiMn-LDH nanoparticles with different Bi content: (a) 10%, (b) 20%, (c) 30%.


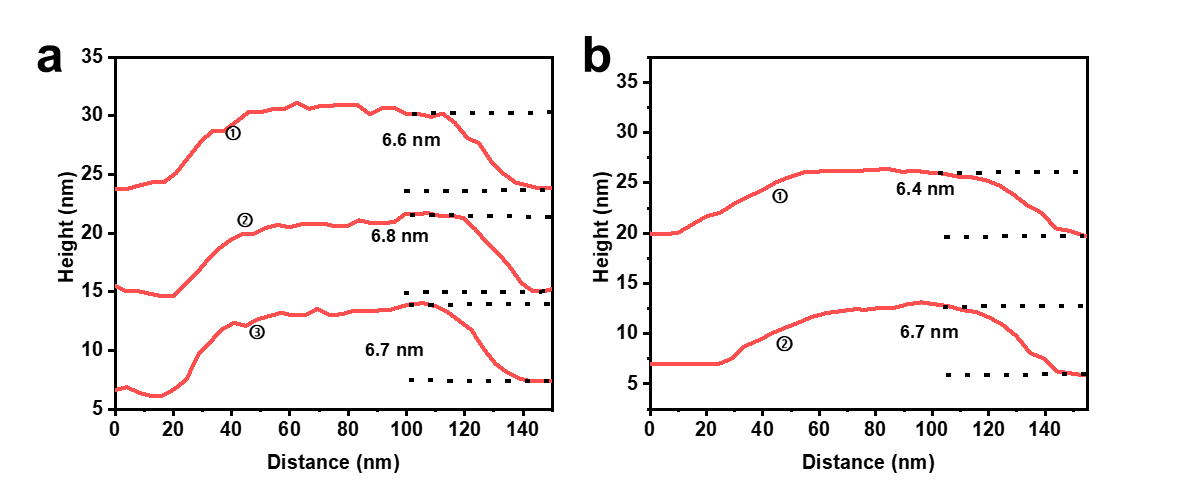


**Fig. S2** AFM height profiles of (a) CoBiMn-LDH and (b) a-CoBiMn-LDH nanoparticles.


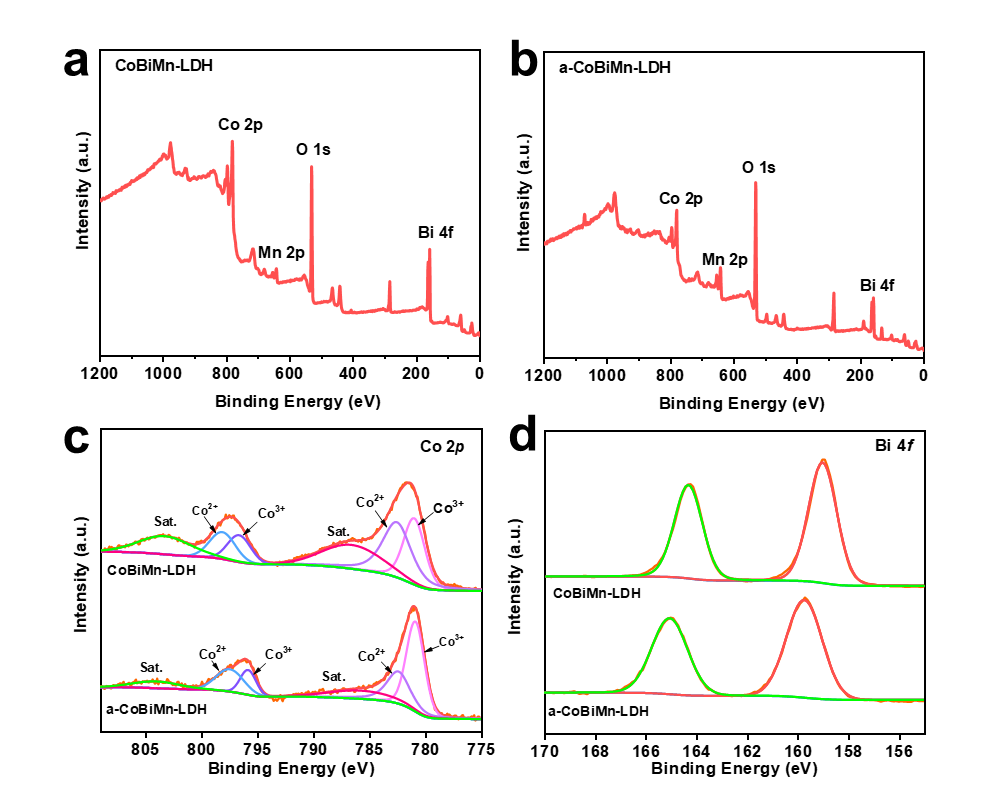


**Fig. S3** Full XPS spectra of (a) CoBiMn-LDH and (b) a-CoBiMn-LDH nanoparticles, and the corresponding (c) Co 2p and (d) Bi 4f spectra.


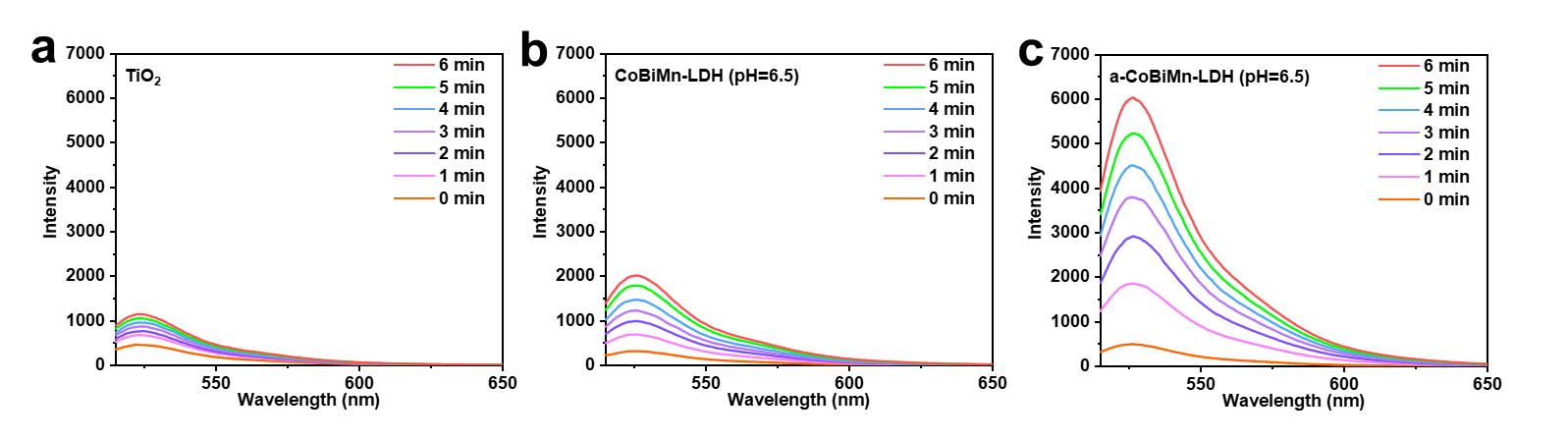


**Fig. S4** Fluorescence spectra of SOSG in the presence of (a) TiO_2_, (b) CoBiMn-LDH and (c) a-CoBiMn-LDH nanoparticles under US irradiation (40 kHz, 3 W cm^−2^) in a buffer solution (pH=6.5).

**
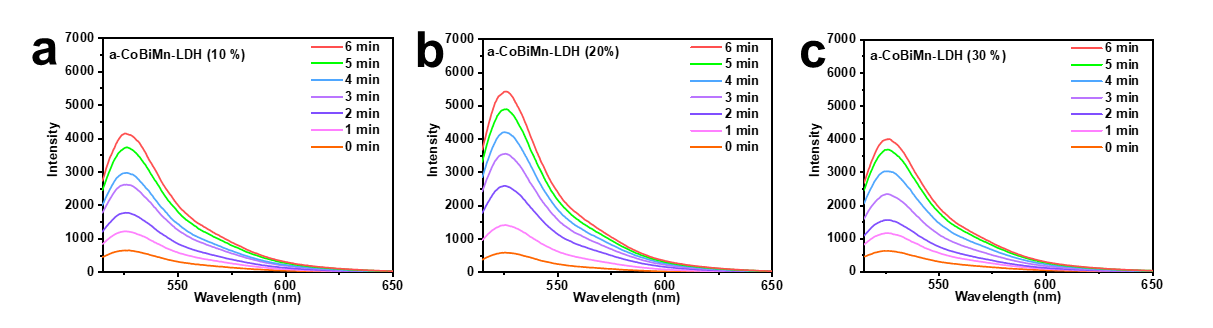
**

**Fig. S5** Fluorescence spectra of SOSG in the presence of a-CoBiMn-LDH nanoparticles with different Bi contents (etched at pH = 4.0 for 10 h) under US irradiation (3 W cm^−2^) in water: (a) 10%, (b) 20%, and (c) 30% Bi.


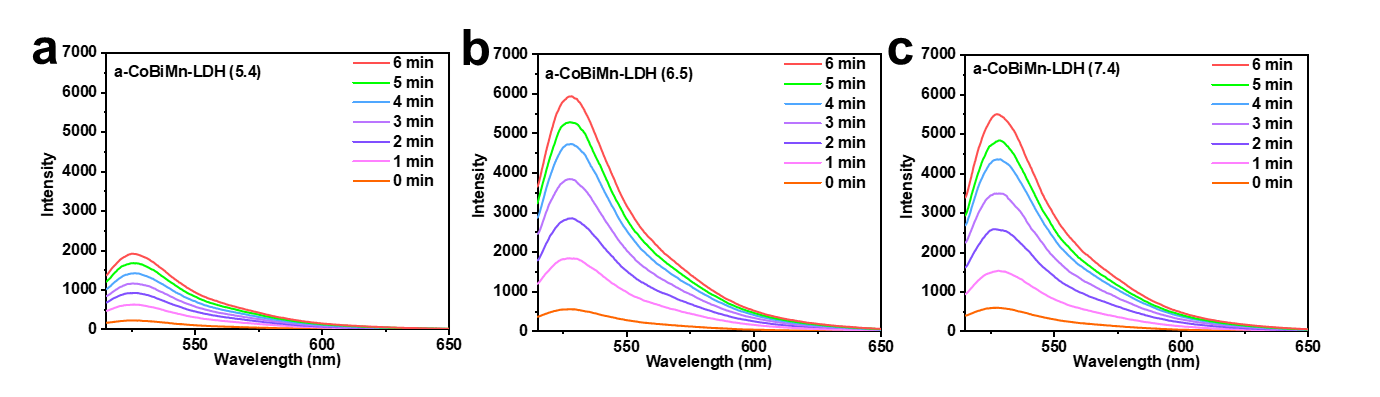


**Fig. S6** Fluorescence spectra of SOSG in different buffer solutions (pH = 5.4, 6.5, 7.4) containing a-CoBiMn-LDH nanoparticles (etched at pH = 4.0 for 10 h) under US irradiation (3 W cm^−2^).


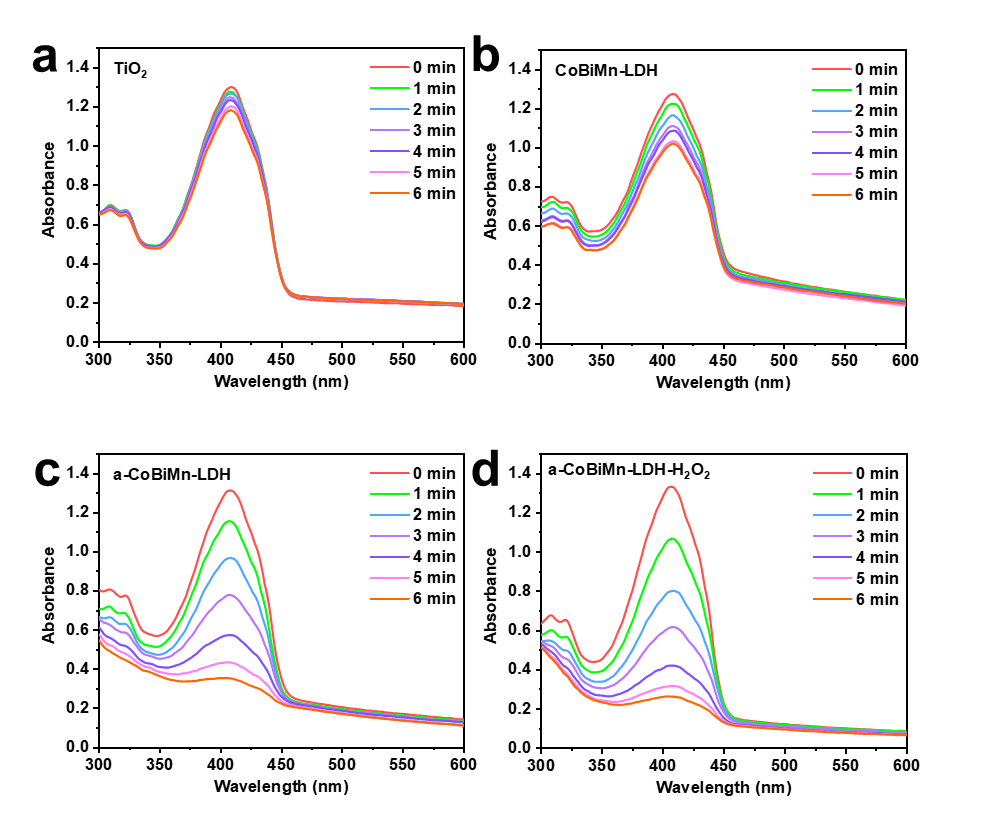


**Fig. S7** UV-vis spectra of DPBF in the presence of (a) TiO_2_, (b) CoBiMn-LDH, (c) a-CoBiMn-LDH and (d) a-CoBiMn-LDH + H_2_O_2_ under US irradiation (40 kHz, 3 W cm^−2^).

**Fig. S8** UV-vis-NIR diffuse reflection spectra of CoBiMn-LDH and a-CoBiMn-LDH nanoparticles.


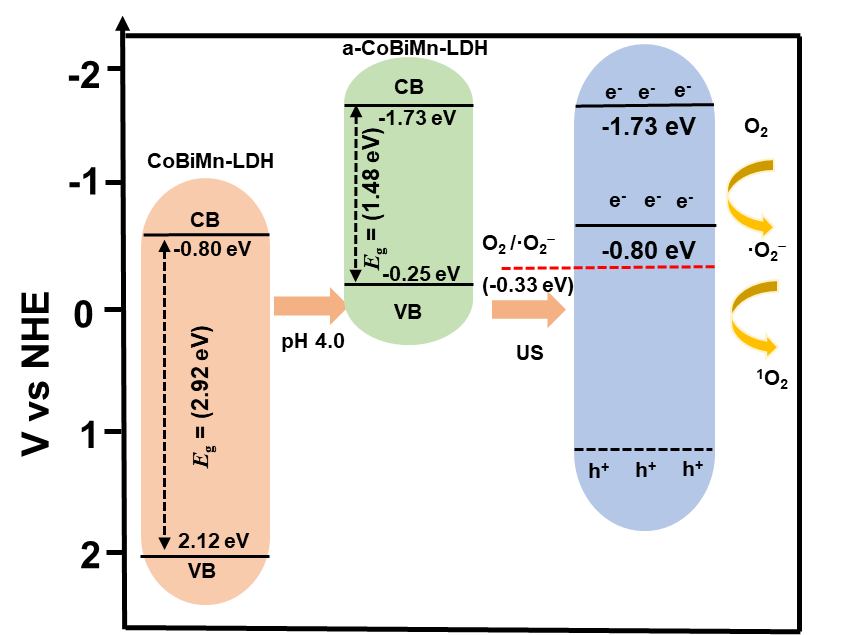


**Fig. S9** Energy level diagrams of CoBiMn-LDH and a-CoBiMn-LDH nanoparticles.


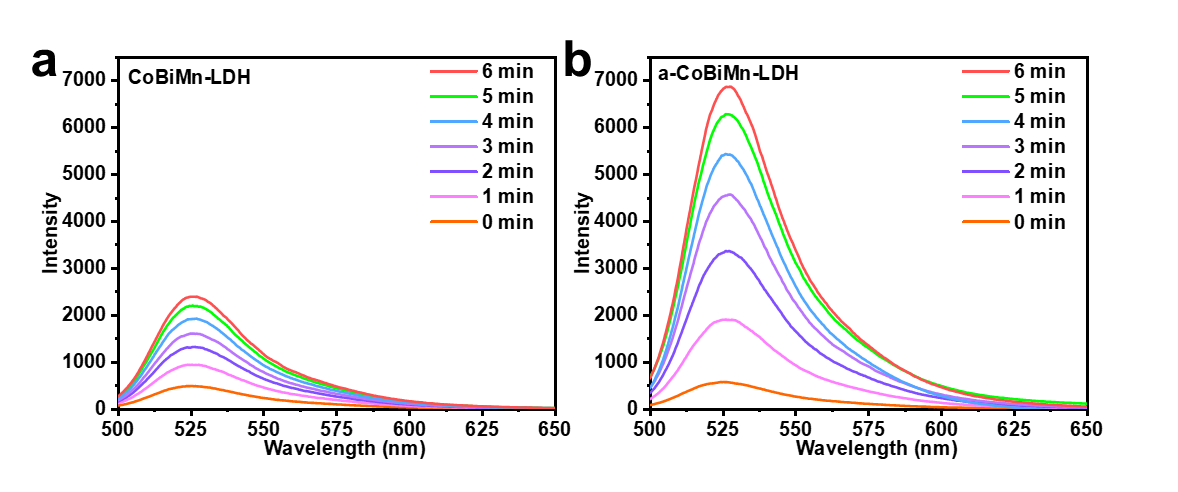


**Fig. S10** Fluorescence spectra of DHR 123 in the presence of CoBiMn-LDH and a-CoBiMn-LDH nanoparticles under US irradiation (40 kHz, 3 W cm^−2^).

**Fig. S11** FT-IR spectra of PEG, CoBiMn-LDH, a-CoBiMn-LDH and a-CoBiMn-LDH-PEG nanoparticles.

**Fig. S12** Zeta potentials of CoBiMn-LDH, a-CoBiMn-LDH and a-CoBiMn-LDH-PEG nanoparticles in water, PBS and DMEM. Data expressed as mean ± S.D. (n = 3).


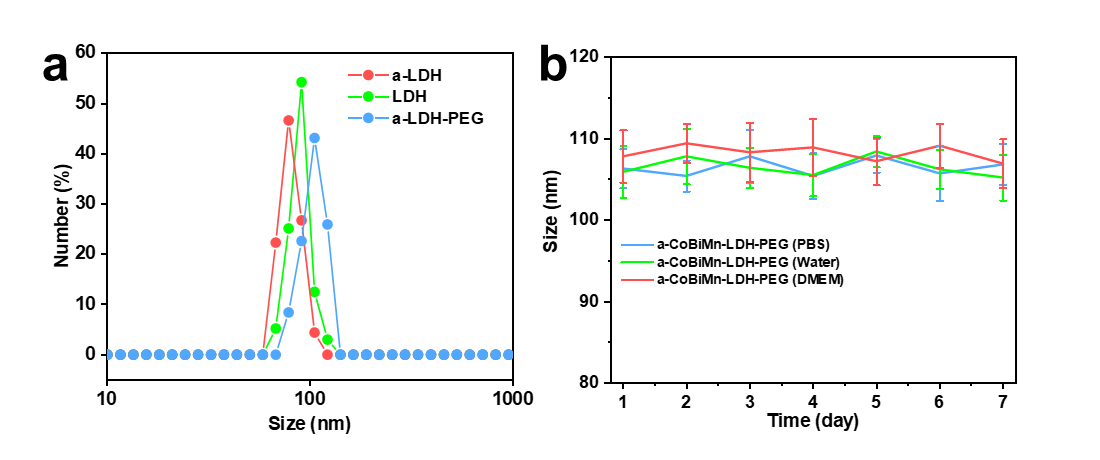


**Fig. S13** (a) Particle size distribution of CoBiMn-LDH, a-CoBiMn-LDH and a-CoBiMn-LDH-PEG nanoparticles. (b) Stability tests of a-CoBiMn-LDH-PEG nanoparticles in water, PBS and DMEM by monitoring its particle size for 7 days. Error bars denote S.D. (n = 3).


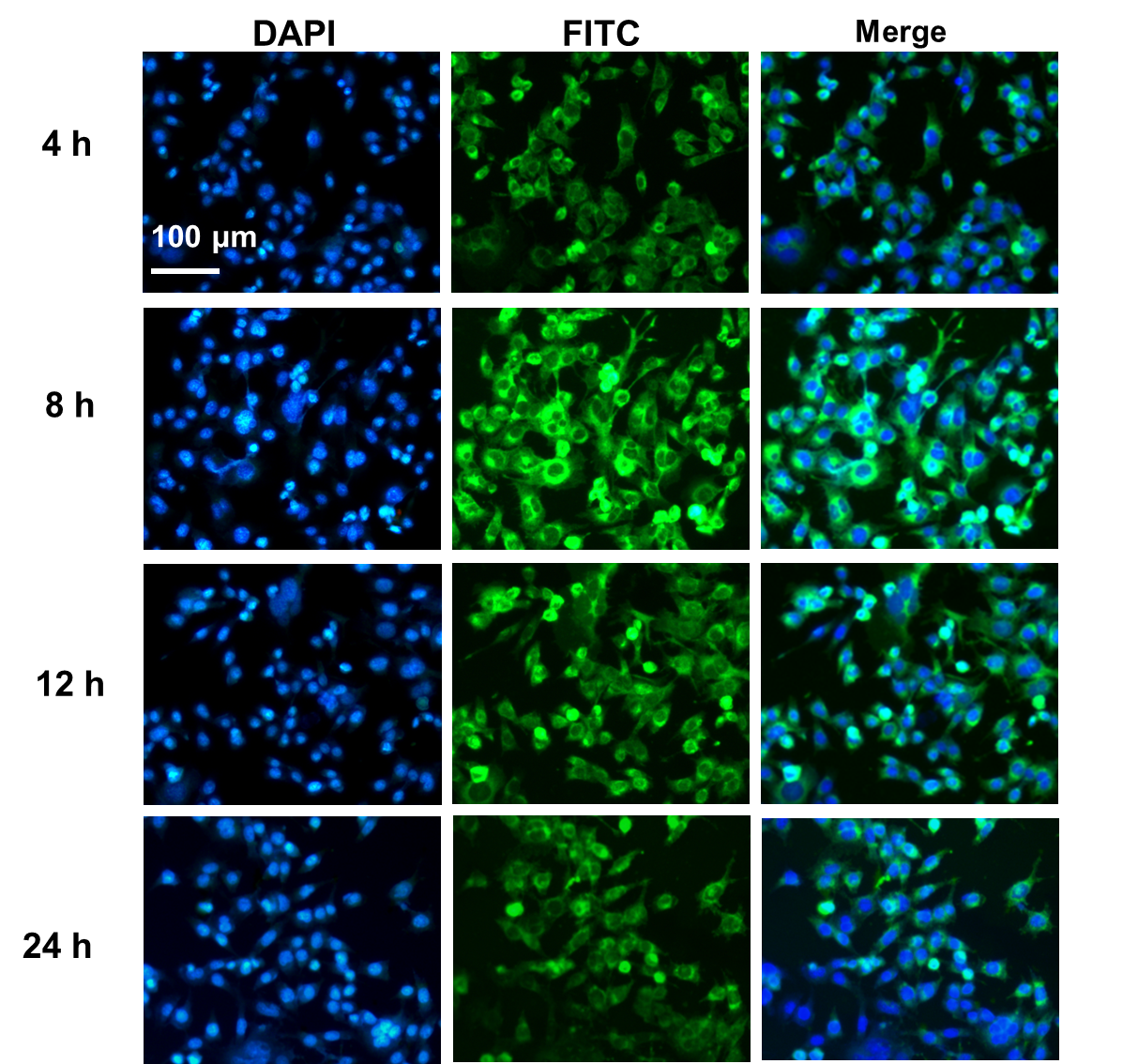


**Fig. S14** Fluorescence images of 4T1 cells treated with FITC-conjugated a-CoBiMn-LDH-PEG for different times (4, 8, 12, and 24 h).


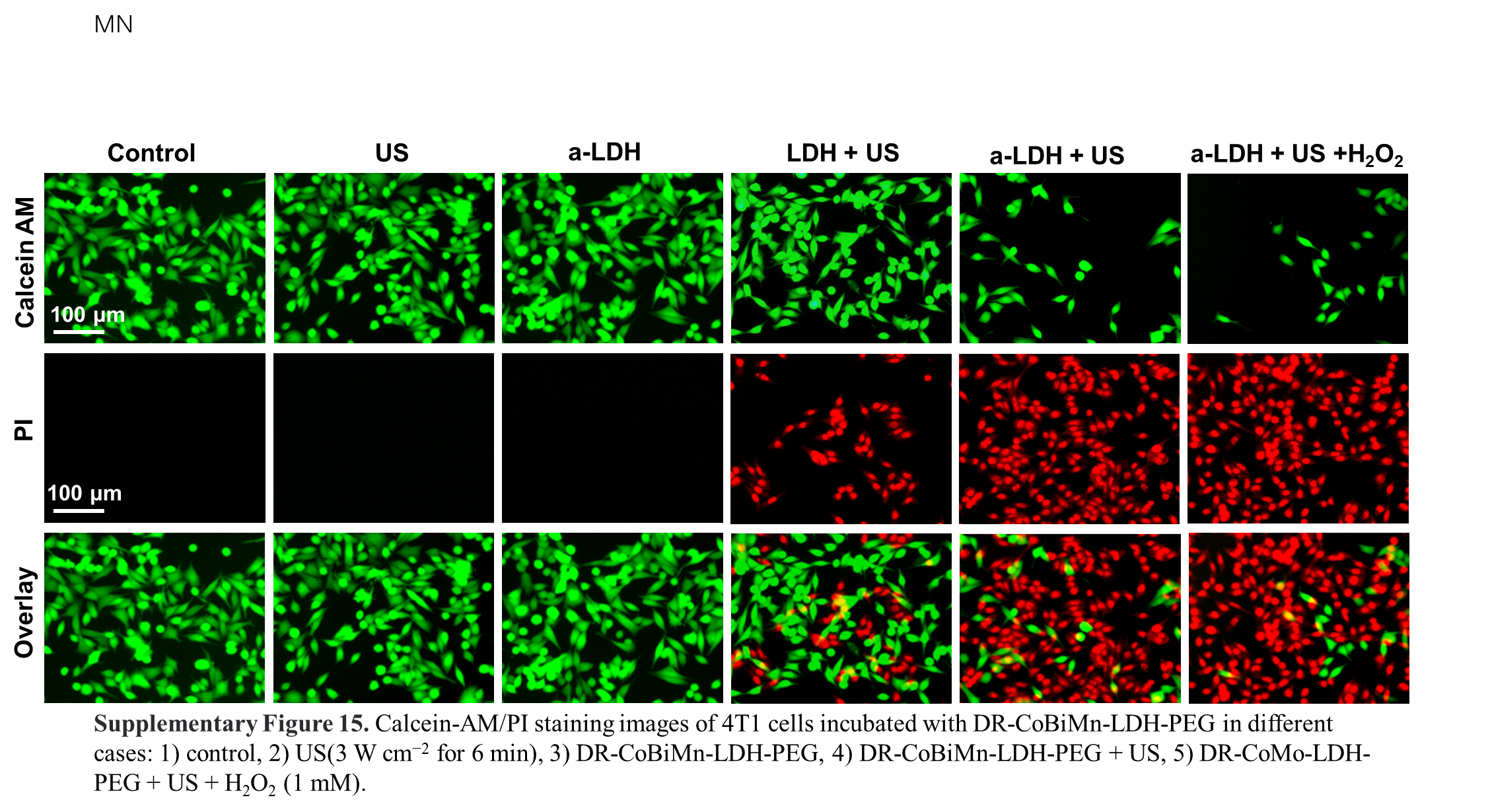


**Fig. S15** Calcein-AM/PI staining images of 4T1 cells after different treatments: 1) control, 2) US (3 W cm^−2^ for 6 min), 3) a-CoBiMn-LDH-PEG, 4) CoBiMn-LDH-PEG + US, 5) a-CoBiMn-LDH-PEG + US, 6) a-CoBiMn-LDH-PEG + US + H_2_O_2_.


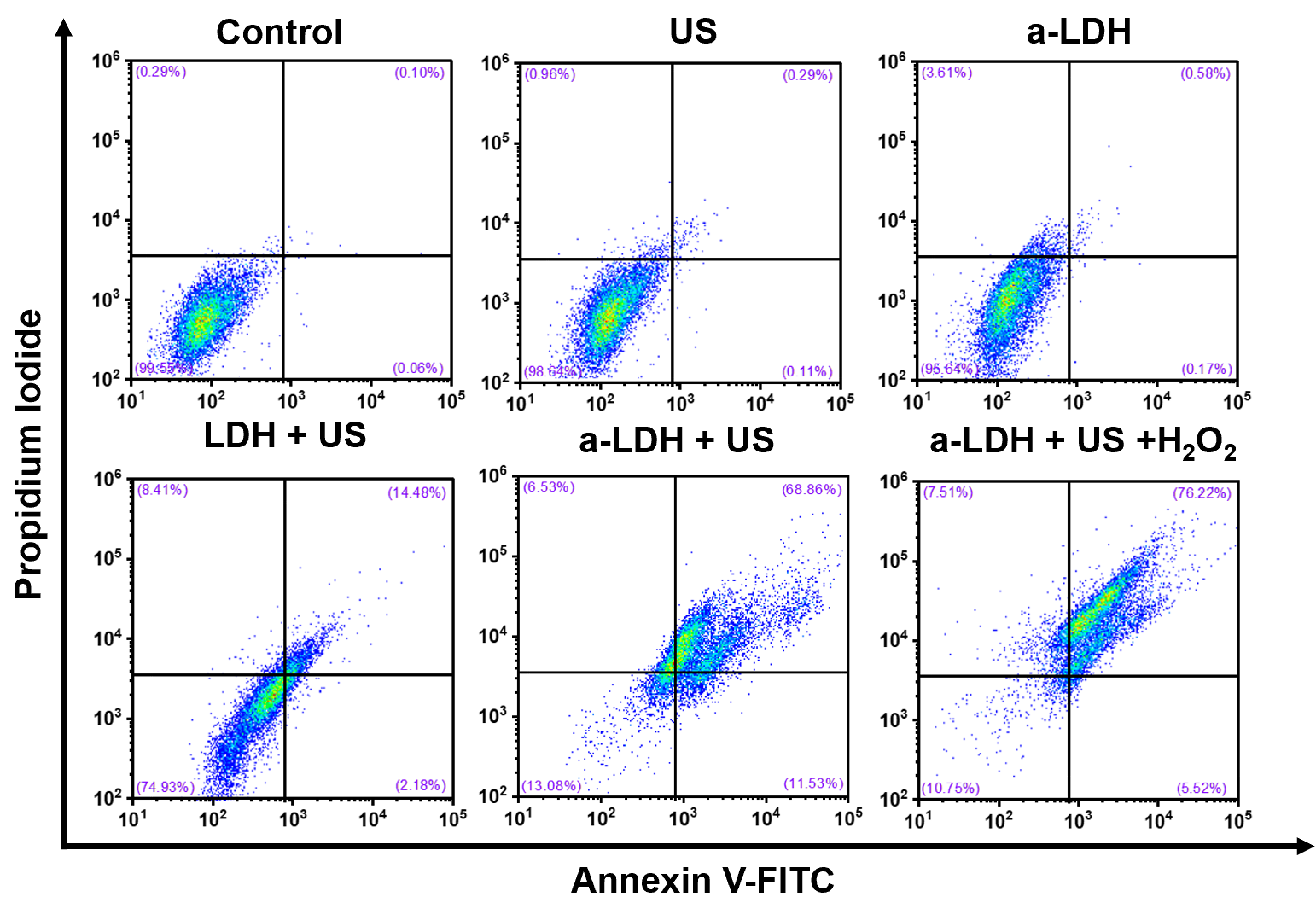


**Fig. S16** Annexin V-FITC/PI double staining analysis of the apoptosis of 4T1 cells after different treatments: 1) control, 2) US (3 W cm^−2^ for 6 min), 3) a-CoBiMn-LDH-PEG, 4) CoBiMn-LDH-PEG + US, 5) a-CoBiMn-LDH-PEG + US, 6) a-CoBiMn-LDH-PEG + US + H_2_O_2_.


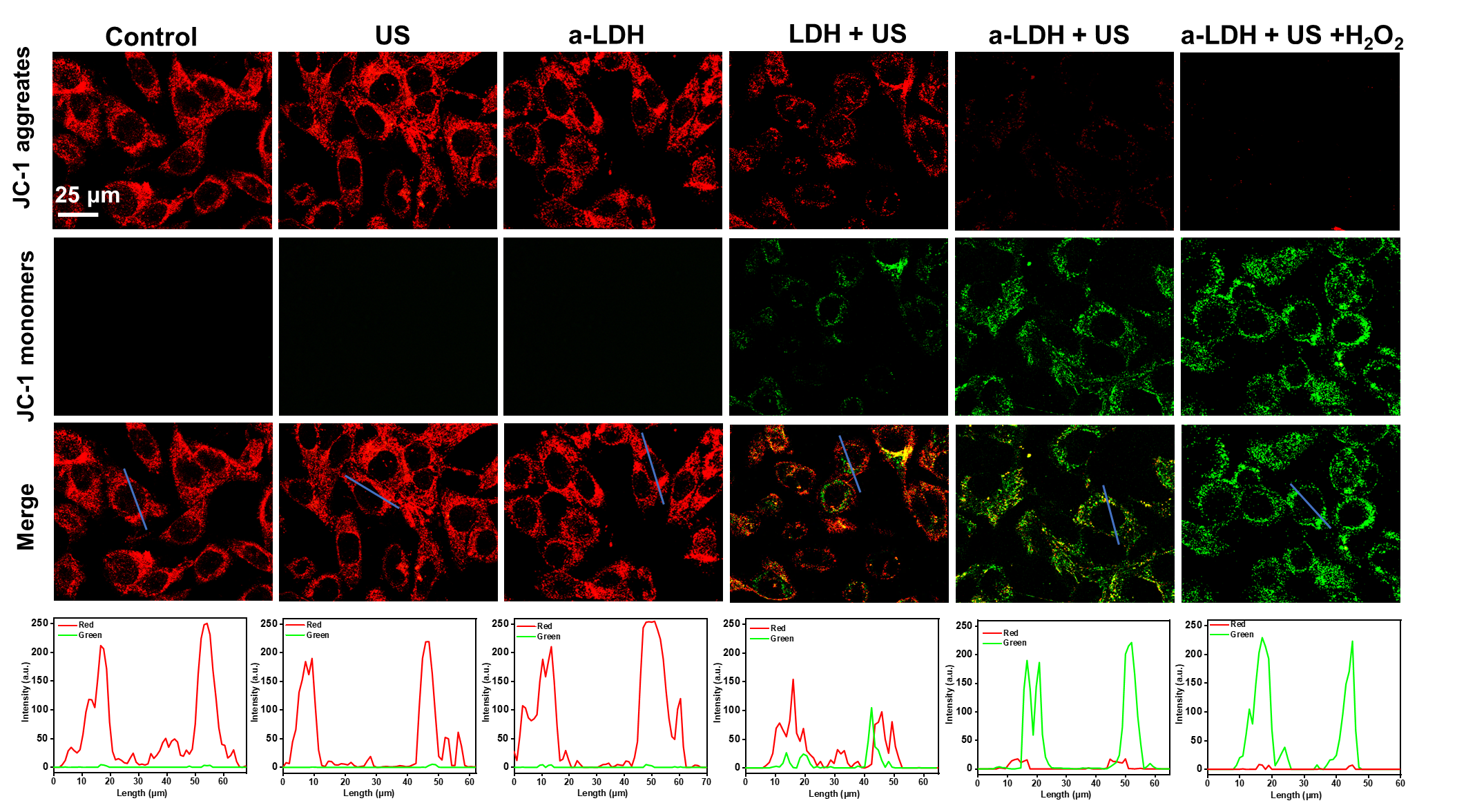


**Fig. S17** JC-1 staining images of 4T1 cells under different conditions: 1) control, 2) US (3 W cm^−2^ for 6 min), 3) a-CoBiMn-LDH-PEG, 4) CoBiMn-LDH-PEG + US, 5) a-CoBiMn-LDH-PEG + US, 6) a-CoBiMn-LDH-PEG + US + H_2_O_2_.

**
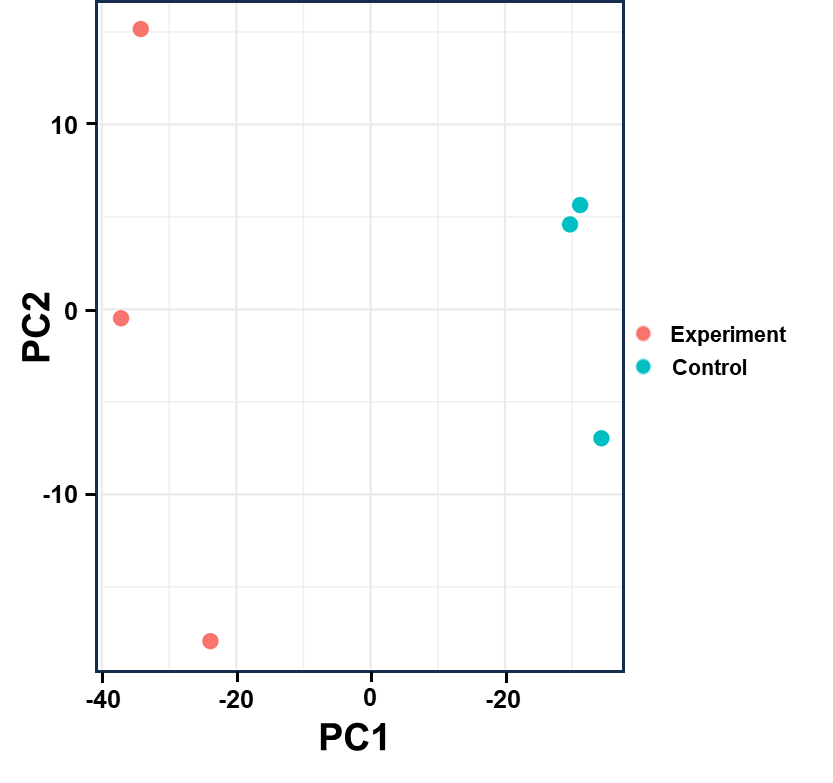
**

**Fig. S18** Principal component analysis of a-CoBiMn-LDH-PEG + H_2_O_2_ + US (experiment) and control groups.


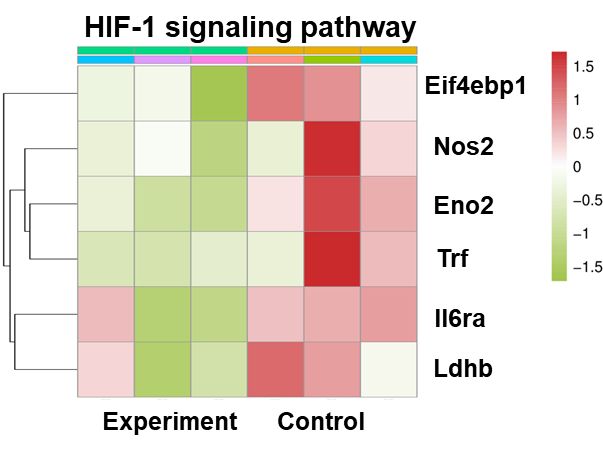


**Fig. S19** Heat map of expressed genes related to the HIF-1 signaling pathway.


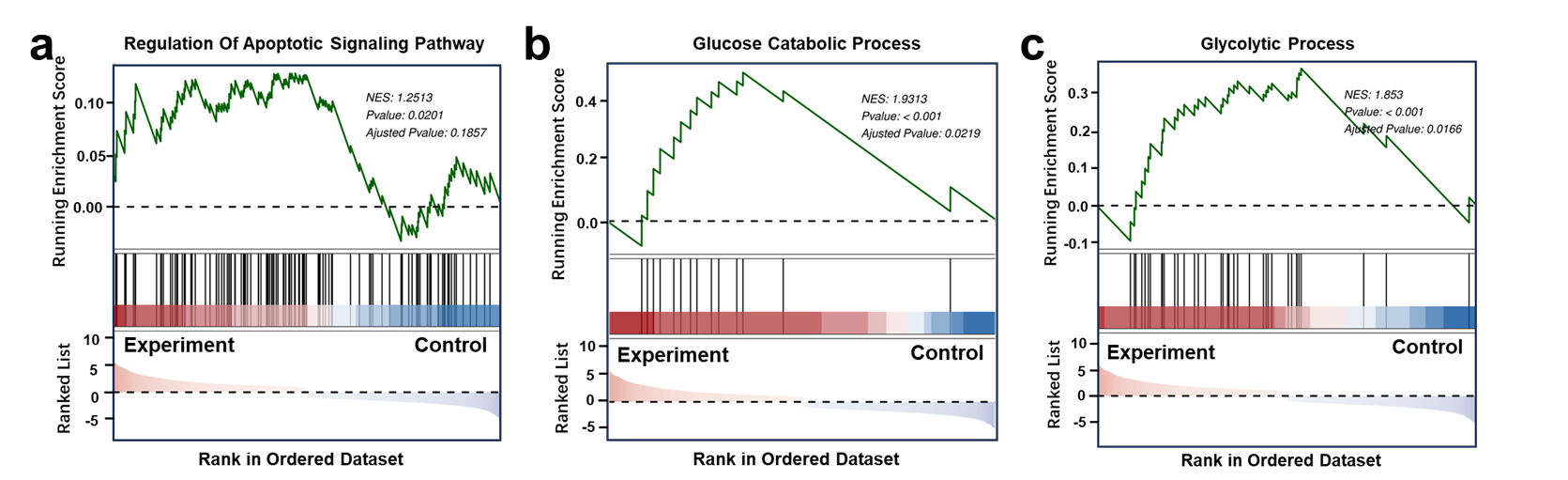


**Fig. S20** GSEA enrichment map of the a-CoBiMn-LDH-PEG + H_2_O_2_ + US group compared to the control group.


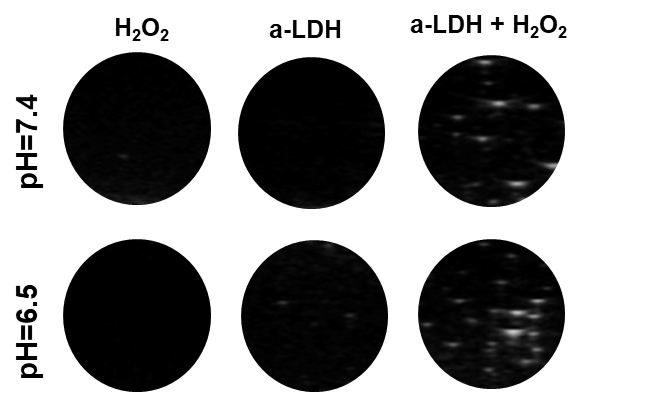


**Fig. S21** *In vitro* US imaging of H_2_O_2_, a-CoBiMn-LDH and a-CoBiMn-LDH + H_2_O_2_.

**Fig. S22** Body weight vs time curves of mice given various treatments. Error bars denote S.D. (n = 6).


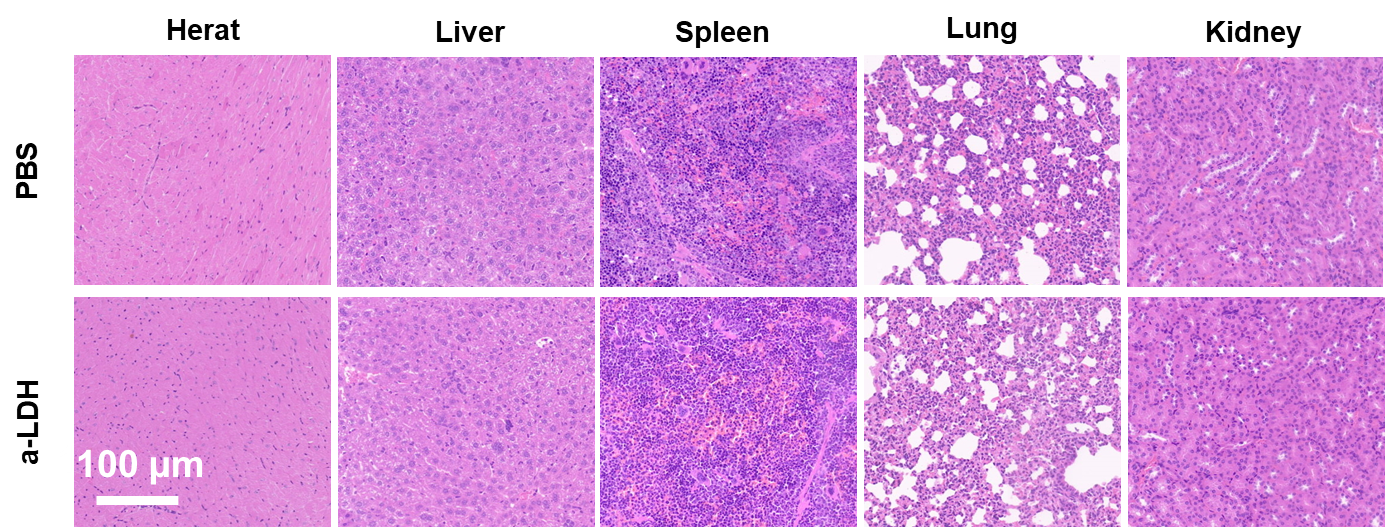


**Fig. S23** Histological images of major organs collected on day 16 after injection of PBS or a-CoBiMn-LDH-PEG.

**Fig. S24** Survival rate of 4T1 tumor-bearing mice after different treatments.

**Fig. S25** Co content in the excreta of mice injected with a-CoBiMn-LDH-PEG nanoparticles (*p < 0.05, **p < 0.01).
